# The impact of Covid-19 lockdown on the gender gap in the Italian labour market

**DOI:** 10.1007/s11150-023-09659-5

**Published:** 2023-06-01

**Authors:** Giulia Bettin, Isabella Giorgetti, Stefano Staffolani

**Affiliations:** 1 Department of Economics and Social Sciences, Marche Polytechnic University, Piazzale Martelli 8, Ancona, Italy; 2 Ismeri Europa, Roma, Italy; 3 GLO—Global Labor Organization, Essen, Germany

**Keywords:** C21, D04, J16, J21, Covid-19, Lockdown, Labour market, Gender gap, Difference-in-differences

## Abstract

We study the gendered impact of the nationwide lockdown (March–May 2020) due to the Covid-19 pandemic on the Italian labour market. Based on Labour Force Survey data on the first three quarters of 2020, we define a Triple Difference-in-Differences (DDD) strategy by exploiting the exact timing of the lockdown implementation. After controlling for several individual and job-related characteristics, we found that in non essential sectors (treated group) the lockdown enlarged pre-existent gender inequalities in the extensive margin of employment: the probability of job loss got 0.7 p.p. higher among female workers compared to their male counterparts, and this difference was mainly detected during the reopening period rather than in the strict lockdown phase. The probability to benefit from the wage guarantee fund (CIG), a subsidy traditionally granted by the government for partial or full–time hours reduction, was also higher for female compared to male treated workers (3.6 p.p.), both during the lockdown and in the reopening phase. This marks a great change with respect to the past, as the application of short-term work compensation schemes was traditionally restricted to male-dominated sectors of employment. On the other hand, no significant gender differences emerged among the treated group either in the intensive margin (working hours) or in terms of remote working, at least in the medium-term.

## Introduction

The shock stemming from the Covid-19 pandemic and related containment measures had major consequences on the world economy that translated into a GDP contraction by 3.3 percent in 2020 (IMF, 2021). Output and employment drops due to the lockdown were everywhere dramatic, far worse than those experienced during the 2007-2009 financial crisis. Compared with pre-pandemic scenarios, in 2020 95 million additional people worldwide fell below the extreme poverty threshold (IMF, 2021). Although extraordinary policy support by national governments and international institutions prevented even worse economic outcomes, the pandemic could likely translate into a persistent increase in economic and social inequalities both within and across countries (Adams-Prassl et al., 2020; Blundell et al., 2020; Shibata, 2020).

One key dimension of these inequalities is gender, because of the unique nature of the labour market shock caused by the pandemic (Alon et al., 2020; Blundell et al., 2020; Oreffice & Quintana-Domeque, 2021). The strict lockdown implemented across the world led to school and daycare closures that massively increased parents’ care duties. Given that mothers provide a much larger share of childcare than fathers usually do (OECD, 2016), such closures mostly affected women’s ability to work, either from the traditional workplace or from home. Moreover, the pandemic recession had its biggest impact on sectors such as hospitality and tourism with high female employment shares, differently from previous recessions that mostly affected sectors such as construction and manufacturing in which men are predominantly employed. For these reasons, the Covid-19 pandemic has often been labelled as “she-cession”, thereby referring to a disproportionate effect on women’s employment (Alon et al., 2022; Bluedorn et al., 2021; Fabrizio et al., 2021). Evidence has been provided also on the fact that women suffered higher non–monetary costs such as worsening well–being, deterioration of mental health and domestic violence (Croda & Grossbard, 2021).

The present paper contributes to the Covid-19-related evidence by analysing the gendered impact of the 2020 lockdown on the Italian labour market. Italy is an interesting case study for several reasons. With respect to the Covid-19 pandemic, it was the first advanced economy to impose an unanticipated lockdown nationwide to all non essential economic activities. Many other Western countries would introduce similar measures only in subsequent weeks and months, as the pandemic was spreading globally. Italian households were forced before anyone else to sudden reconcile school and daycare closures with the unexpected shift to remote working in jobs that could somehow be performed from home, and with essential activities that stayed open despite the high risk of contagion (e.g. health sector). Up to the pandemic, Italy had one of the lowest smart/remote working rates across Europe: only 10% of workers worked from home several times a week, compared to an average 16% in the EU-27 (Eurofound, 2021). During the lockdown, almost 40% of Italian workers had to start working from home.

The lockdown effects on the Italian labour market are worth investigating also due to structural low levels of female labour force participation and employment. Despite the progresses observed in the last two decades,^1^ the female employment rate was only slightly above 50% at the beginning of 2020, the second lowest value across the whole European Union. Existing literature has also well documented the existence of a large and persistent gender wage gap (Addabbo & Favaro, 2011; Brown et al., 2022; Casarico & Lattanzio, 2023; Cascella et al., 2022; Mussida & Picchio, 2014; Piazzalunga & Tommaso, 2019) as well as significant costs for working women due to motherhood in terms of both career break job penalties and wage penalties (Casarico & Lattanzio, 2021; Pacelli et al., 2013; Picchio et al., 2021). This situation is mostly due to the existence of social norms and stereotypes (Alesina & Giuliano, 2013), such as the traditional male breadwinner model, that are deep-rooted in the Italian society (Istat, 2019) and strongly influence female involvement in paid work (Anxo et al., 2011; Barigozzi et al., 2020, 2022). Italy is the EU country where women spend the most time in upaid activities (Campaña et al., 2023) and where gender differences over time dedicated to unpaid work are the highest (Istat, 2019). It is also a consequence of inadequate parental leave and child care policies (Brilli et al., 2016; Carta, 2019; del Boca & Wetzels, 2008) and of the still limited availability of educational services for early childhood, especially in Southern regions (Istat, 2022). Stereotypes are also likely to affect students’ outcomes and their choice of the field of study (Carlana, 2019). Indeed, most Italian women still discard the selective and highly rewarding S.T.E.M. fields to concentrate in the less employable, low paying majors of humanities. This horizontal segregation is a substantial component in men–women earnings gap during their early career (Anelli & Peri, 2015) but tends also to persist over time (Bianco et al., 2013).

Against this background, our aim is to shed light on the immediate labour market consequences of the nationwide lockdown imposed by Italian government between March and May 2020, and how they differed between male and female workers. The lockdown policy determined economic activities that had to stop, whereas those deemed essential (health care, food service, and public transportation, among others) could operate and were urged to maintain social distancing measures to the extent possible.

By using Labour Force Survey (LFS) data on the first three quarters of 2020, we investigate the consequences of the lockdown policy on four labour market outcomes: job loss, hours worked per week, access to the wage guarantee fund (CIG), and remote working.^2^ The wage guarantee fund called “Cassa Integrazione Guadagni” (CIG) is a short-term work compensation scheme that firms may use to preserve employment relationships in case of suspension or reduction in working activities due to temporary events that cannot be ascribed directly to the firm itself. Compared to the standard pre-Covid scenario, the requirements to access such compensation schemes were relaxed by law during the lockdown and CIG application greatly expanded.

Our Triple Difference-in-Differences (DDD) strategy (Angrist & Pischke, 2009; Gruber, 1994; Imbens & Wooldridge, 2008; Lechner, 2011; Olden & Møen, 2022; Wooldridge, 2010) is based on the exact timing of the lockdown implementation by the Italian government, on the classification of economic sectors as essential or non–essential and on gender, in order to analyse whether and how gender inequalities emerged among workers employed in sectors that were locked down (treated workers). We further distinguish the effects due to the strict shutdown from those related to the reopening phase of non-essential economic activities, in order to understand better the persistency of the effects induced by the policy shock. The use of LFS data allow to control for several individual and job-related characteristics.

Results show that in non essential sectors (treated group), the probability of job loss got 0.7 p.p. higher for females, and the differentiated impact on the extensive margin of employment was mainly detected during the reopening period rather than in the strict lockdown phase. The probability to benefit from the wage guarantee fund (CIG) was also higher for female compared to male workers (3.6 p.p.) in the treated group, both during the lockdown and in the reopening phase. This is a direct consequence of the policy intervention that expanded short-term work compensation schemes, whose application up to the 2020 lockdown was traditionally restricted to male–dominated sectors of employment. No significant gender differences were instead detected either on the intensive margin, in terms of working hours, or in terms of remote working, at least in the short–term.

Some evidence has already been provided regarding the gendered impact of the lockdown on the Italian labour market: Casarico and Lattanzio (2022) used administrative data on the first two quarters of 2020 and focussed on contracts dynamics’ in terms of hirings and separations (layoffs, endings of fixed-term contracts and quits). Compared to this work, besides assessing the effect on the extensive margin in terms of job losses, we look at the intensive margin by analysing wheter women adjusted their working hours differently from men in response to the pandemic. Moreover, we provide a direct test of gender differences in the access to the wage guarantee fund (CIG), which were assessed only indirectly by Casarico and Lattanzio (2022) through their (expected) impact on hirings and separations.

The set-up of our paper is as follows. In the next section, we briefly review the recent literature on the gendered impact of Covid-19 emergency in the labour market, with a specific focus on the Italian context. Section 3 describes the Covid-19 emergency and the implementation of public policies by the Italian government after March 2020. Section 4 explains the econometric model and the identification strategy whereas data are presented in section 5. Section 6 reports and comments on estimation results, falsification checks, heterogeneity and robustness. Section 7 concludes. An additional online Appendix provides further descriptive statistics, the full set of estimation results as well as validity and robustness checks.

## The gendered impact of Covid-19

### International evidence

In several countries, the decline in either employment and/or hours worked due to the Covid-19 pandemic had been larger among women compared to men (Alon et al., 2020). However, pre-Covid19 situation as well as policy interventions played an important role in shaping heterogeneous effects across countries: Alon et al. (2022) showed that the gender gap was substantial in the response of hours worked in Canada, Germany and the US. Small differences in the intensive margin were instead registered in the Netherlands, Spain, and the United Kingdom. Other studies stated that the gender gap worsening was observed along the extensive rather than the intensive margin, due either to the relative decline in female labour force participation (Bluedorn et al., 2021) or to a relative rise in female job losses (Adams-Prassl et al., 2020; Dang & Nguyen, 2020; Galasso & Foucault, 2020; Montenovo et al., 2020). The evidence, however, is not conclusive, with contrasting results emerging even on the same country based on different data sources. As far as UK is considered, for example, Hupkau and Petrongolo (2020) found that the incidence of job losses and furloughing was similar between males and females, but average female losses on the intensive margin were slightly smaller, as in Bluedorn et al. (2021). Andrew et al. (2020) instead showed that working mothers were more likely to lose their job or be furloughed during the lockdown and among those who were still working, time spent on paid work decreased whereas time spent on childcare increased.

Country studies on either the US or European labour markets mostly focussed on workers/households with dependent children and intra–household gender differences in the impact of the lockdown due to the asymmetric increase in childcare responsibilities due to school closures (Sevilla & Smith, 2020). Mothers were the hardest–hit both along the intensive (Amuedo-Dorantes et al., 2020; Zamarro & Prados, 2021) and the extensive margin of participation in paid employment (Fabrizio et al., 2021; Heggeness, 2020).

Whereas the cross-country evidence provided by Bluedorn et al. (2021) showed that the she–cession experienced in the second quarter of 2020 faded, in most cases, by the following quarter, Albanesi and Kim (2021) estimated a slightly longer persistence of the effects on US female employment and labour force participation: women accounted for 70% of the total decline in participation rates in spring 2020, and up to 100% in the fall.

### The impact on the Italian labour market

As far as Italy is concerned, the existing evidence on the gendered consequences of the lockdown on either the extensive or the intensive margin of labour market participation is still relatively scant.

Several contributions focussed on the adoption of remote/smart working practices and their impact on labour market outcomes, given that Italy up to the pandemic had one of the lowest incidence rates of remote working across Europe (Eurofound, 2021). Barbieri et al., (2022) classified occupations according to the possibility to work from home and found that in essential sectors that were not forced to close (e.g. service sector) the risk of contagion had been mitigated by working remotely. Depalo and Giorgi (2021) showed that the increase in the incidence of remote working was larger among female workers, and compared to men they also experienced larger benefits from remote working in terms of monthly wages, hours worked and access to redundancy funds (CIG). Along the same line, Aina et al. (2021) looked at the effects of COVID–19 pandemic on the wage distribution by means of quantile regressions based on LFS data up to the second quarter of 2020, and found that women would be the major beneficiaries from the long run increase in the possibility to work from home. On the contrary, the evidence provided by Bonacini et al. (2021a), by means of Oaxaca-Blinder decomposition and unconditional quantile regressions based on the INAPP-PLUS 2018 survey, showed that the pandemic crisis would have negative long-term implications for women, given that the pre–Covid gender wage gap was greater in occupations with a high level of work from home attitude, and in particular among older and married female employees. In other words, according to their results the “new normal” of working from home would exacerbate pre-existing inequalities in the labour market by favouring male, older, high–educated, and high–paid employees (Bonacini et al., 2021b).

Some additional works investigated the impact of Covid–19 on the intra–household division of non–paid housework and childcare, and thus provided only an indirect assessment of the labour market consequences of the pandemic. Data collected on a representative sample of working and non–working Italian women showed that the gender gap in the household division of unpaid labour widened during both the first and the second wave of Covid-19 (Del Boca et al., 2020, 2021). The evidence provided in Mangiavacchi et al. (2021) instead supports the idea that the lockdown had a balancing effect on the parental division of household tasks, with a significantly larger contribute of fathers to childcare and homeschooling activities. However, this effect was strongly dependent on parents’ employment status during lockdown. Fathers performed more household tasks if they were at home alone with their children whereas the opposite happened if mothers stopped working.

To the best of our knowledge, a direct assessment of the lockdown impact on gender differences in labour market outcomes was provided only by Casarico and Lattanzio (2022). They employ Italian administrative data on a sample of active contracts up to the second quarter of 2020 to look at the change in weekly hirings and terminations relative to the corresponding average in 2017–2019. According to their results, there was a pronounced drop in hirings starting with the introduction of the lockdown measures. On the contrary, a sharp increase was observed in layoffs and quits up to the introduction of the firing freeze policy (see Subsection 3.2), after which both layoffs and quits dropped significantly. By estimating a cross-sectional linear probability model for job loss which mainly controls for individual-level characteristics, they showed that young, temporary and low–skilled workers experienced a greater reduction in the separation probability with respect to the pre-Covid scenario, thus suggesting that policy measures were effective in protecting these categories during the lockdown. Female workers instead experienced lower benefits from the policy intervention compared to their male counterparts, as they were more likely to separate from their job immediately after the pandemic kicks in.

## Policy intervention

### The lockdown implementation

In Italy, the first cases of Covid-19 were detected at the end of January 2020, but the spread of the disease accelerated only in the second half of February and Lombardia was the epicentre of the outbreak. Two local “red zones” involving 11 municipalities in the provinces of Lodi and Padua were implemented on February 22 whereas the first nation-wide measure was announced and signed by the Prime Minister on March 4 and became effective the day after. It concerned mainly the suspension of school activities at any grade from kindergarten to university.

On March 8, with 5800 confirmed cases and 233 deaths, the Italian government signed a restriction act that extended the quarantine zone to the entire Lombardia region and to other 14 provinces in North and Central Italy,^3^ thus affecting over 16 million residents. Travel from, to or within the affected areas were restricted, funerals and cultural events were banned and a one-metre minimum distance between people was imposed in all public places. Restaurants and cafes could only work between 6 am and 18 pm whereas many other places such as gyms, swimming pools, bars, museums were closed. Firms and offices were asked to implement remote working whenever possible to limit contagion. This measure had to become effective the day after, although the contents of the decree had already been anticipated in the media the day before the signature. On the evening of 9 March, however, the Prime Minister announced that the quarantine measures would be extended to the entire country from March 10.

On March 11, after two weeks in which the number of worldwide cases outside China had a 13-fold increase and the number of affected countries tripled, the World Health Organization declared that Covid-19 could be characterized as a pandemic. The following day, with the virus spreading exponentially across the country, the Italian government tightened the national lockdown measures. All commercial and retail economic activities were closed down, apart from those providing essential goods and services (grocery stores, food stores, pharmacies). Even cafes and restaurants were closed with the exception of take-away services. People were allowed to exit home only to go to work, to do grocery shopping and for emergency reasons.

Due to a dramatic rise in the number of cases and deaths, local autorities, trade unions and also public opinion called for a generalised shutdown including all non-necessary businesses and industries. A decree established essential economic activities that could continue to operate and non–essential activities that were forced to shut down according to Ateco 2007 classification of economic activities.^4^ Essential sectors included agriculture, some manufacturing, energy and water supply, transports and logistics, banking and insurance, information and communication activities, professional and scientific activities, public administration, education, healthcare and few service activities. On the other hand, shutdown activities included most of manufacturing, wholesale and retail trade, hotels, restaurants and bars, entertainment and sport activities. After March 25 only few sectors remained fully operative and up to 3 million workers inside these sectors (for example in finance and insurance, professional services as well as public administration) were working from remote (Barbieri et al., 2022). We consider the 11th week (March 9th–15th) as the start of the national lockdown (Casarico & Lattanzio, 2022).

The so-called “Phase 2” was announced by the Italian government on April, 26 and the nation-wide lockdown expired on May 4. Since then, manufacturing and construction resumed their activities under new safety rules (staggered shifts, temperature checks, masks), but retail shops, cafes, restaurants, services (hairdressers, beauticians, gyms, swimming pools) and touristic activities were still closed and reopened on May 18 although with some flexibility across regions. Sports facilities reopened on May 25, followed by cinemas and theatres on June 15. Mobility across regions was still forbidden until June 3, whereas people were allowed to move across municipalities for work and health reasons as well as for visiting relatives. Since most of the activities reopened by May 25, this is the official end of the lockdown period in our empirical setting.

**Fig. 1:**
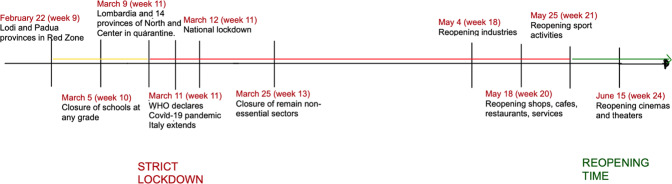
The lockdown in Italy: implementation timeline

Figure 1 summarises the timeline of the lockdown implementation, and the way we deal with it in our empirical strategy. We considered the start of lockdown in week 11 (in red), as in Casarico and Lattanzio (2022), with the exceptions of two provinces, Padua and Lodi, for which the policy intervention started two weeks earlier (in yellow). The reopening time (in green) started from week 21 (May, 25) when most of the activities in non-essential sectors reopened. The weekly frequency is adopted for three out of four outcomes in our analysis: working hours, CIG benefit and remote working. For the job loss outcome, we have a monthly time basis, as explained in Subsection 5.1. The lockdown then started from March whilst the reopening period ran from June. In our empirical specification, we also control for time-fixed effects by fixing the starting week (month) to 0 and rescaling the calendar weeks (months) in relative weeks (months).

### Additional labour market and social protection measures

On March 17, the government adopted a €25 billion emergency package (Law Decree No. 18/2020, the so–called “Cura Italia” Decree), which included also specific measures to increase workers’ protection.^5^ A ban on individual and collective dismissals was initially introduced from 17 March 2020 for 60 days and has been extended several times until 30 June 2021. The freeze was related to all layoffs opened after February 23rd, including those for economic reasons. The natural expiry of the fixed–term contracts remained out of scope.

The same package extended the use of the “Cassa Integrazione Guadagni”, thereafter CIG, a short-term work compensation scheme normally granted by the government in case of suspension or reduction in working activities due to temporary events that cannot be ascribed to the company. The subsidy is replacing 80% of the forgone earnings due to working hours reduction, up to a predetermined threshold. The Covid19-related CIG was initially provided for a maximum of 9 weeks between 23 February and 31 August 2020, further prolonged by the Relaunch Decree released on May 19 in order to preserve employment relationships while cutting firms’ labour costs during the lockdown period. This measure was extended to firms with less than 15 employees, usually excluded from its ordinary application, and to firms using the extra–ordinary CIG, one of the subcatefories of CIG that usually cannot be cumulated with the ordinary one. One-off bonuses were introduced for self-employed workers, professionals and (mostly temporary) workers in the touristic, entertainment and agricultural sector.

Remote working was strongly recommended in the private sector from the beginning of the emergency, and according to the provisions in the “Cura Italia” Decree it became the regular working method for most of the public sector during the pandemic. The use of agile/remote working by the employer was authorised even in the absence of any agreement between the employee and the employer which is normally required. Disabled workers were granted the right to work from remote, if their tasks were consistent with teleworking. And so were working parents employed in the private sector with children under the age of 14, provided that the other parent was still working.

The “Cura Italia” Decree also introduced additional specific measures in order to help working parents to face both school closures and the impossibility to rely on grandparents’ informal childcare during the lockdown. Private and public sector employees and self-employed workers with children up to 12 years old were granted a special parental leave up to 15 days during the period of school closure, with an indemnity equal to 50% of their compensation. Moreover, workers with children between 12 and 16 years old could benefit from an unpaid leave, during which they could not be dismissed. Such parental leave was granted provided that the other parent was not unemployed or granted with similar measures. As an alternative to parental leave, parents with children up to 12 years old were entitled to apply for a 600-euro one-off bonus for babysitting services during lockdown.

## Methodology

### Identification strategy and empirical modelling

The aim of our empirical analysis is to assess whether and how the Covid-19 lockdown affected the labour market outcomes of workers in non-essential economic sectors, with a specific focus on gender differences.

In general, let *y*_*i*,*p*,*t*_ be the outcome of interest of individual *i* who resides in province *p* at time *t*.^6^ We define *S*_*i*,*t*_ as a binary variable equal to 1 if worker *i* is employed in a non-essential economic sector (*treatment group*) and 0 in an essential one (*control group*) at time *t*, according to what described in Section 3. *C*_*i*,*p*,*t*_ is another binary variable equal to 1 if the information collected from worker *i* living in the province *p* refer to a post-lockdown period,^7^ and 0 otherwise. Finally, the dummy variable *fem*_*i*_ refers to the gender of worker *i* (1 female, 0 male).

The identification of the effect of the lockdown by gender on our outcomes of interest is based on a *Triple Difference-in-Differences* (DDD) approach with repeated cross-sections (Angrist & Pischke, 2009; Gruber, 1994; Imbens & Wooldridge, 2008; Lechner, 2011; Olden & Møen, 2022; Wooldridge, 2010).

We set up the following linear regression model:

1 $$\begin{array}{lll}{y}_{i,p,t}\,=\,{\delta }_{0}+{\delta }_{1}{S}_{i,t}+{\delta }_{2}{C}_{i,p,t}+{\delta }_{3}{S}_{i,t}\times {C}_{i,p,t}+{\delta }_{4}fe{m}_{i}+{\delta }_{5}{S}_{i,t}\times fe{m}_{i}\\ \qquad\qquad+\,{\delta }_{6}{C}_{i,p,t}\times fe{m}_{i}+{\delta }_{7}{S}_{i,t}\times {C}_{i,p,t}\times fe{m}_{i}+{x}_{i,t}^{{\prime} }{\gamma }_{1}+{z}_{p,t}^{{\prime} }{\gamma }_{2}+{\varepsilon }_{i,p,t},\end{array}$$

where *S*_*i*,*t*_ × *C*_*i*,*p*,*t*_ × *fem*_*i*_ is the triple treatment and *δ*_7_ is the gendered average treatment effect on the treated (gATT), our coefficient of interest. Such point estimate is unbiased because it calculates the time change in means for women employed in the treated group by netting out both the change in means for women in the control group and the change in means for men employed in the treated group. In other words, a DDD design ensures the consistency of gATT estimations by exploiting the triple differences and thereby removing all the confounding trends both within gender, between essential and non essential workers, and across gender, in the treated subsample of non essential workers. In particular, as stated in Wooldridge (2010, p.151) this identification strategy accounts for two kind of potentially confounding trends: changes across sectors due to gender status and unrelated to the lockdown implementation, and changes in labour market outcomes of workers employed in the essential sectors possibly due to sector-specific changes in the economy (e.g. workers switching from non-essential to essential sectors) that affect all workers, regardless of gender. Furthermore, this identification strategy also accounts for the criticism to the credibility of the traditional Difference-in-Differences models within the Covid-19 pandemic raised by Weill et al. (2021). Indeed, it would be very hard to exclude that the lockdown had any impact, albeit small, also on the control group, and this would be troublesome in the conventional DiD setting, thereby translating into non-robust estimations to minor specification changes (Weill et al., 2021).^8^

*x*_*i*,*t*_ contains exogenous or pre-treatment individual characteristics at time *t*, such as citizenship, age cohorts, level of education, number of children by age cohort, employment status (employee, self-employed, etc.), years of experience and tenure until 2019, worker qualification (ISCO-08 at 1 digit) and its own index of remote working, sector of activity, firm size, and the pre-treatment share of females workers expressed at 3-digit sectoral level. These information can be considered as exogenous or pre-determined because the pandemic and the subsequent lockdown acted as a sudden and unexpected shock on the labour demand side,^9^ also because Italy was the first European country to experience a rapid and dramatic increase in contagion. To capture the different speed with which the virus spread throughout the country, we also add a set of covariates *z*_*p*,*t*_ that includes a proxy for the intensity of contagion per week at the province level, together with time^10^ and province fixed effects. The inclusion of weekly/monthly fixed effects help to control for seasonality patterns we may observe in our labour market outcomes whereas sector fixed effects together with the pre-treatment share of females workers at 3-digit sectoral level could account for sectoral specificities which might generate gender differential seasonality. The parameters of Eq. (1) are estimated by Ordinary Least Squares (OLS) with standard errors clustered by sectors at 4-digit level as a simple way to deal with correlation within-groups (Liang & Zeger, 1986). For further details on the interpretation of the estimated coefficients, see Section A in the Online Appendix.

Finally, to test the robustness of our baseline results, we augment the DDD treatment-effects estimation with the kernel propensity-score matching (PSM) following Heckman et al. (1998, 1997), and Blundell and Costa Dias (2009),^11^ as well as with the inverse probability treatment weighting (IPTW), as in Stuart et al. (2014) and Austin and Stuart (2015). More specifically, in order to account for the potential selection bias caused by workers’ observable characteristics, we estimate a PSM model and then a DDD method for mitigating the problem of selection by unobservable characteristics. Results are presented in Section 6.4.

### Assumptions validity

The OLS estimations of the DDD model in Eq. (1) require some assumptions hold in order to return unbiased estimates of the causal effect of the lockdown implementation.

The first assumption is related to the parallel trend and states that, conditional on the control variables, treated individuals (employed in non-essential economic sectors subject to lockdown) would have followed similar trends in the labour market outcomes as non treated individuals (employed in essential economic sectors not subject to lockdown) in the absence of the intervention, distinguishing by gender. This assumption is not directly testable because we cannot observe the counterfactual evolution of the outcomes, but, however, it can be supported by testing whether female and male workers in the two groups were following parallel trends before the lockdown started. In the same spirit of Autor (2003), we checked this by estimating an event study model which includes the leads of the indicator for the lockdown implementation, up to 10 weeks (2 months for job loss estimations) and the lags from 11 to 41 weeks (from 3 to 9 months for job loss). This model is estimated first by splitting the sample by gender and then in the full sample across gender. If the treated and the non treated group by gender as well as the female and male treated group in the full sample experienced parallel trends before the policy implementation, the coefficients of these leads should be nil. This assumption holds during the lockdown implementation, as shown in Subsection 6.2.

The second assumption is related to the exogeneity of the timing of the policy implementation. As described in Section 3, the timing of lockdown implementation is exogenous as it was caused by the rapid spread of the Covid-19 emergency on the national territory. The Italian government quickly implemented the shutdown measures for the whole country to limit the increasing risks of contagion.

The third assumption regards the absence of any anticipation effect in the policy implementation. This assumption would fail if individuals themselves had anticipated the lockdown measure and decided to close their activities before the actual implementation. To assess whether anticipation might be an issue, in Subsection 6.2 we provide a robustness check by removing all individuals interviewed before the implementation, from 6th to 10th week.

## Data and sample

The empirical analysis is based on the Italian Labour Force Survey (LFS) conducted by the Italian Institute of Statistics (ISTAT) during the first three quarters of 2020. The dataset contains individual-level information on current and past work experiences (employment status, characteristics of the main job, unemployment spells, job search, etc.), together with socio-demographic variables. We focus on individuals aged 20–69 which were employed in the week before the interview (“reference” week) or in the month to which the reference week belongs (see Subsection 5.1 for the exact definition of the samples).

Our estimation strategy exploits the information on the reference week and the province of residence kindly provided by ISTAT^12^ in order to set up a Triple Difference in Differences (DDD) design in which we distinguish: (i) the period before and after the policy implementation (lockdown); (ii) two group of workers, those employed in non-essential economic sectors (*treatment group*) and those employed in essential economic sectors (*control group*), as explained in Section 3; (iii) male and female workers. It is worth noting that the Italian LFS provided detailed information on the week of the interview only in 2020, whilst for the previous years the exact timing of the interview inside each quarter was not available. If this information was essential to check for the exact timeline of the lockdown, the fact that it was released only in 2020 prevented us from using data from previous years to build the control group, and from exploiting alternative approaches to investigate the gendered impact of Covid-19 lockdown, such as the event study carried out by Casarico and Lattanzio (2022).

### Outcomes and their dynamics

We look at four different outcomes: job loss, hours worked per week, wage guarantee fund (CIG), and smart/remote working.

Job loss is a dichotomous variable equal to 1 if the individual had lost her/his job in the current month (month to which the reference week belongs) and 0 otherwise. To investigate this outcome, our selected sample (*sample*_1_, 132055 *obs*) includes people who were employed or lost their job in the current month. For this sample, we converted the reference week on a monthly basis, given that information on the last job are available only at monthly level.

Hours worked per week derive from the workers’ self-declaration regarding the number of hours actually worked in the reference week and are a continuous variable (*sample*_2_, 121744 *obs*).^13^

The variable referring to the wage guarantee fund, CIG, is equal to 1 if the individual has benefited from the wage guarantee measure in the reference week and 0 otherwise, given that no information is available on the monetary amount received by each worker. For this outcome, we restrict the sample to employed people who belong to industries that could access this social security measure during the Covid-19 emergency as discussed in Subsection 3.2 (*sample*_3_, 67,368 *obs*).^14^

The last outcome refers to remote working; it is equal to 1 if the individual worked remotely for at least one day in the reference week or in the three weeks before, and 0 otherwise. It is computed for the same sample of dependent employees used for the wage guarantee measure (*sample*_3_) in order to focus the analysis on the private sector and have a better balance between the treated and the control group.^15^

For each sample, Table 1 shows the distribution of frequencies across treatment (non-essential sectors) and control (essential sectors) groups, both before and after the lockdown implementation (for additional details, see Table B.1 in the Online Appendix). Table 2 reports the unconditional mean for all the outcome variables across treatment and control groups, both before and after the lockdown implementation (for more details, see Table B.2 in the Online Appendix).

**Table 1:** Samples—Frequencies by groups

	Sample 1	Sample 2	Sample 3
	Job loss	Working hours	CIG/Remote working
All			
Before, Treated	10,971	9074	6432
Before, Control	21,183	17,718	8615
After, Treated	33,623	31,627	22,257
After, Control	66,278	63,325	30,064
Total	132,055	121,744	67,368
Males			
Before, Treated	6965	5749	3956
Before, Control	10,887	9112	4890
After, Treated	21,476	20,205	13,955
After, Control	33,814	32,182	16,905
Total	73,142	67,248	39,706
Females			
Before, Treated	4006	3325	2476
Before, Control	10,296	8606	3725
After, Treated	12,147	11,422	8,302
After, Control	32,464	31,143	13,159
Total	58,913	54,496	27,662

Sample 1 for job loss includes people who are employed or have lost their job in the reference month. Sample 2 for working hours is limited to individuals employed in the reference week. Sample 3 for CIG and remote working considers individuals employed in the reference week and is restricted to employees only. It further excludes those working in sectors which could not benefit from the wage guarantee fund (CIG): agriculture, forestry and fishery, public administration, defence, education, human health and social work activities, extra-territorial organisations and bodies

The treated group includes workers in non-essential sectors, whilst the control group represents workers in essential sectors

**Table 2:** Outcomes—Unconditional means by groups

	Job loss	Working hours	Cig	Remote working
	Sample 1	Sample 2	Sample 3	Sample 3
All				
Before, Treated	0.006	36.522	0.007	0.012
Before, Control	0.005	34.461	0.002	0.024
After, Treated	0.013	27.300	0.143	0.070
After, Control	0.002	29.894	0.056	0.135
Total	0.006	30.379	0.073	0.088
Males				
Before, Treated	0.005	39.061	0.009	0.012
Before, Control	0.004	38.485	0.002	0.025
After, Treated	0.010	29.489	0.131	0.061
After, Control	0.002	33.612	0.056	0.128
Total	0.005	33.499	0.071	0.080
Females				
Before, Treated	0.008	32.133	0.004	0.011
Before, Control	0.006	30.201	0.001	0.023
After, Treated	0.018	23.426	0.161	0.086
After, Control	0.002	26.053	0.056	0.144
Total	0.006	26.528	0.075	0.099

Sample 1 for job loss includes people who are employed or have lost their job in the reference month. Sample 2 for working hours is limited to individuals employed in the reference week. Sample 3 for CIG and remote working considers individuals employed in the reference week and is restricted to employees only. It further excludes those working in sectors which could not benefit from the wage guarantee fund (CIG): agriculture, forestry and fishery, public administration, defence, education, human health and social work activities, extra-territorial organisations and bodies

The treated group includes workers in non-essential sectors, whilst the control group represents workers in essential sectors

**Fig. 2:**
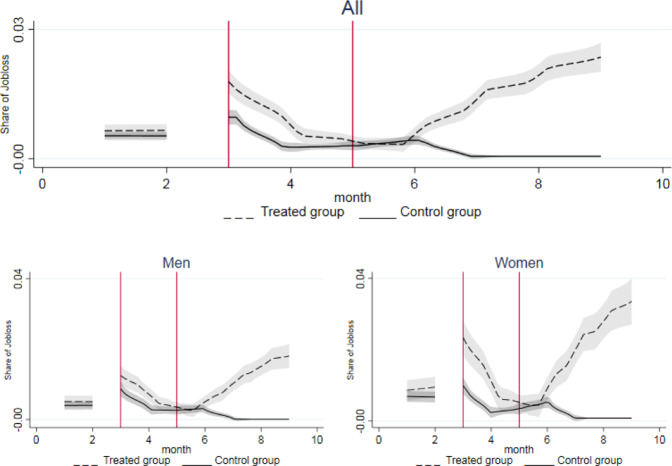
Trend of Job Loss by groups. The x axis describes the first three quarters of 2020 in months. Starting from the left, the first break (first vertical line) marks the beginning of the lockdown in March, the second break (second vertical line) marks the end of the lockdown and the period of reopening of economic activities in May. Confidence interval at 5% in grey. The treated group includes workers in non-essential sectors, whilst the control group represents workers in essential sectors

**Fig. 3:**
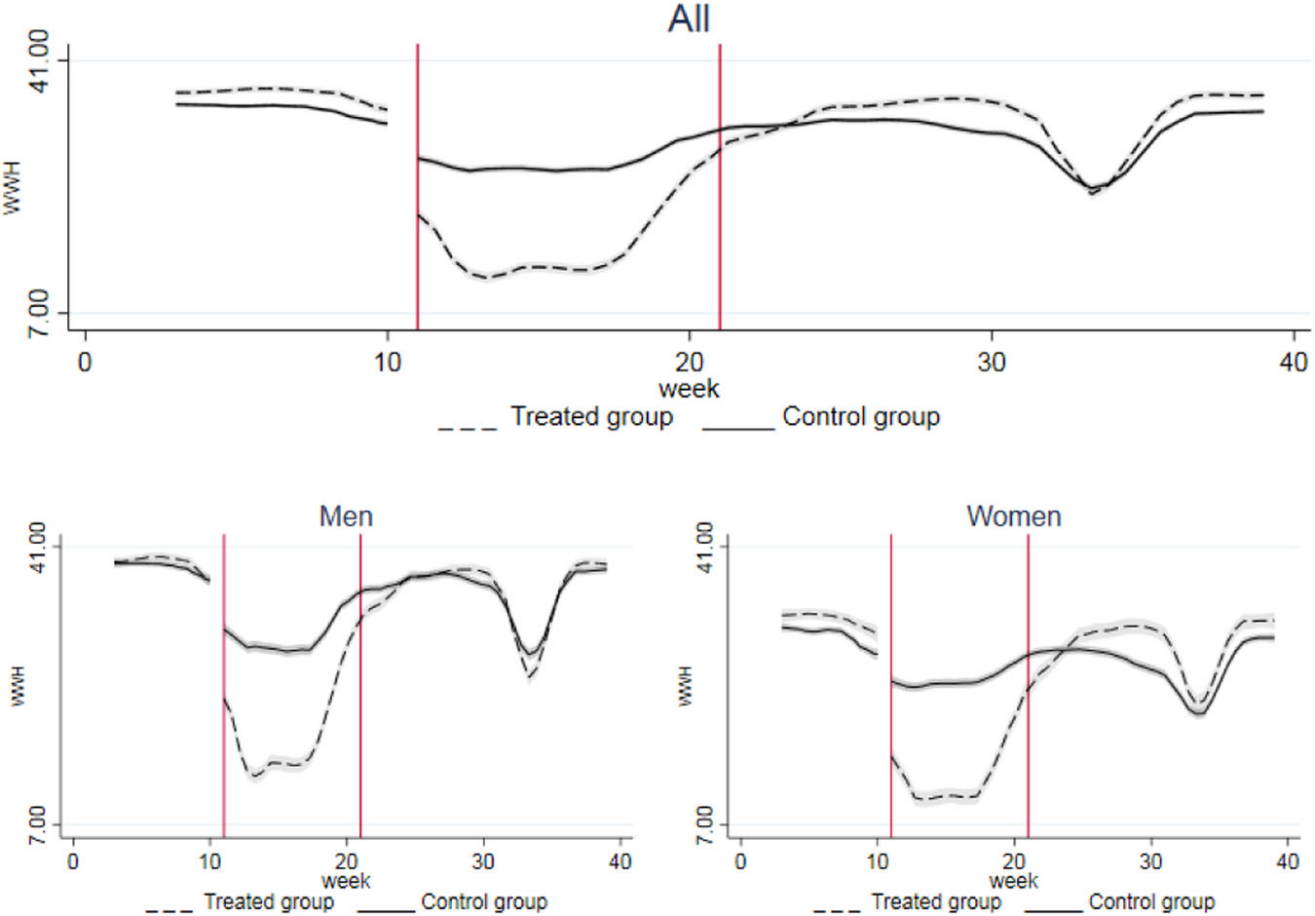
Trend of weekly working hours by groups. The x axis describes the first three quarters of 2020 in weeks. Starting from the left, the first break (first vertical line) marks the beginning of the lockdown at week 11, the second break (second vertical line) marks the end of the lockdown and the period of reopening of economic activities at week 21. Confidence interval at 5% in grey. The treated group includes workers in non-essential sectors, whilst the control group represents workers in essential sectors

**Fig. 4:**
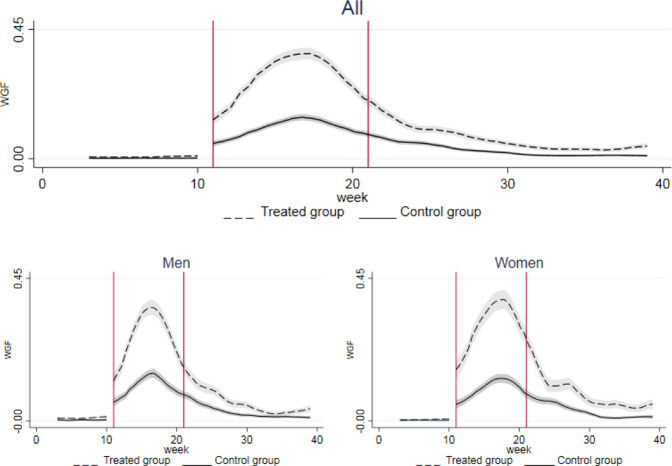
Trend of wage guarantee fund (CIG) by groups. The x axis describes the first three quarters of 2020 in weeks. Starting from the left, the first break (first vertical line) marks the beginning of the lockdown at week 11, the second break (second vertical line) marks the end of the lockdown and the period of reopening of economic activities at week 21. Confidence interval at 5% in grey. The treated group includes workers in non-essential sectors, whilst the control group represents workers in essential sectors

**Fig. 5:**
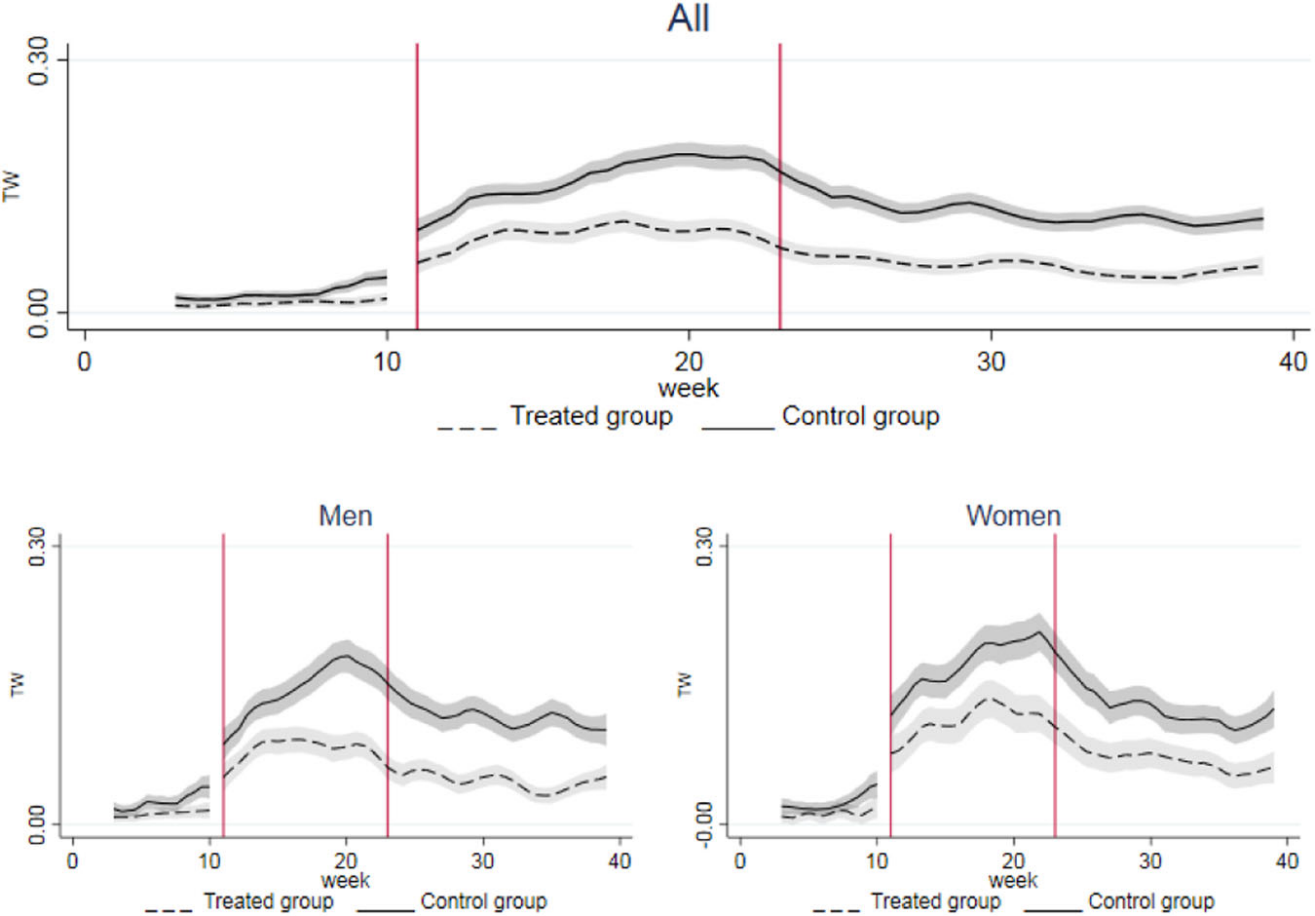
Trend of remote working by groups. The x axis describes the first three quarters of 2020 in weeks. Starting from the left, the first break (firs vertical line) marks the beginning of the lockdown at week 11, the second break (second vertical line) marks the end of the lockdown and the period of reopening of economic activities at week 21. Confidence interval at 5% in grey. The treated group includes workers in non-essential sectors, whilst the control group represents workers in essential sectors

From the beginning of the lockdown (March 2020 for job loss, 11th week for working hours, CIG and remote working), a significant discontinuity is detected in all our outcomes. Figures 2–5 show their dynamics over time. We can observe two shocks in the period: the first one in March with the lockdown implementation and the second one in June when economic activities reopened (the so called Phase-2). Even though such shocks are detected also in the full sample, when disaggregating by gender they look more intensive for female workers.

In particular, the probability of losing the current job more than doubled for the treated group after the lockdown (from 0.006 to 0.013), and it had a steeper increase for treated women (from 0.008 to 0.018). This trend was confirmed also in the reopening phase, with a stronger increase in the probability of job loss for women compared to men (Fig. 2).

Working hours per week decreased across all groups, but the decline in the treatment group was far larger (9 vs. 5 h, respectively); however, no specific trend by gender is detected (Table 2). Figure 3 shows that the drops in working hours were recorded during the lockdown (from the 11th to 21st week) and during summer holidays (from the 31st to the 34th week). After the reopening (from the 21st week onwards) the recovery in terms of hours worked was more intense for females than for men.

The wage guarantee fund (CIG) was implemented both in the treated and in the control group after the lockdown. The increase, however, was larger for the treated group. When splitting the sample by gender, similar trends were observed in the control group whereas in the treated one the incidence among women was twice that among men, although female workers had a lower probability of benefiting from it in the period before the lockdown, due to a more extensive use of this instrument in sectors such as manufacturing and construction, with a traditionally lower share of female employment. LFS data showed that after the global financial crisis women accounted for less than one third of workers benefiting from the CIG, 27,7% in 2009 and 29% in 2010, respectively (Istat, 2011). This incidence was indeed in line with the share of female workers in the manufacturing sector in 2010 (27,2%). Even at the end of our period of analysis, the use of CIG was still larger compared to the situation before the lockdown, especially for women (Fig. 4).

Finally, the probability of remote working increased in both essential and non-essential economic sectors after the lockdown, with a major prevalence recorded among women. The incidence of remote working rose, respectively, by almost 8 times among treated females and by more than 6 times among untreated ones. Even after the end of the lockdown, the use of remote working was substantial compared to the pre-lockdown scenario (Fig. 5).

### Other covariates

The covariates considered in the model refer to demographic and household characteristics, as well as job and firms specifics.^16^

In particular, demographic and household characteristics include citizenship status (1 foreign citizenship, 0 Italian citizenship), age categories (20–24, 25–29, 30–34, 35–39, 40–44, 45–49, 50–54, 55–59, 60–64, and 65–69 years), level of education (none, primary education, secondary education, and tertiary or more), number of children by age category (0–5, 6–10, 11–15 years old).

Job and firm characteristics include the status of being an employee (vs. self-employed and/or professional), years of experience, years of tenure and the nature of the contract (part-time vs. full-time and temporary vs. permanent job).^17^ Furthermore, we control for the type of occupation (ISCO08 code at 1 digit), the type of industry (ATECO/NACE code at 1 digit),^18^ the size of the firm (less 10 employees, 11–15, 16–19, 20–49, 50–249, more than 250 employees),^19^ and the share of female workers employed in each sector at 3-digit level before the lockdown implementation.^20^

In our estimated specification, we also include a composite index based on the ICP survey by the National Institute for Public Policies Analysis (INAPP)^21^ in the same spirit of Barbieri et al. (2022). Such *remote*_*index* proxies for the feasibility of a remote working arrangement, ranges from 0 to 100 and is computed by taking the average of the following seven questions: (i) importance of performing general physical activities (which enters with reversely); (ii) importance of working with computers; (iii) importance of manoeuvreing vehicles, mechanical vehicles or equipment (reversely); (iv) requirement of face-to-face interactions (reversely); (v) dealing with external customers or with the public (reversely); (vi) physical proximity (reversely); (vii) time spent standing (reversely).^22^In order to capture the different speed of epidemics spreading at the local level, we also include the variable “*positive*_*pop*” built as the ratio between the weekly number of people who tested positive for Covid-19 in each Italian province and the total resident population in the same province on 1 January 2020.^23^ In the estimated model, it enters with a lag of one week.

Finally, we also control for fixed effects in terms of province of residence and of “relative weeks”^24^ from the start of lockdown (or “relative months”, for job loss estimation). A list of the main covariates used in the estimated model and their descriptive statistics are reported in Table 3.

**Table 3:** Descriptive statistics of the main covariates

	Sample 1		Sample 2		Sample 3	
	Job loss		Working Hours		CIG/Remote working	
	Mean	Std. Dev.	Mean	Std. Dev.	Mean	Std. Dev.
Female	0.446	0.497	0.448	0.497	0.411	0.492
Foreign citizenship	0.132	0.339	0.132	0.339	0.170	0.375
Age cohorts:						
- 20–24	0.005	0.070	0.005	0.070	0.007	0.083
- 25–29	0.040	0.195	0.039	0.193	0.056	0.230
- 30–34	0.063	0.244	0.063	0.243	0.078	0.268
- 35–39	0.083	0.275	0.082	0.275	0.094	0.292
- 40–44	0.105	0.306	0.105	0.307	0.116	0.320
- 45–49	0.134	0.341	0.134	0.341	0.141	0.348
- 50–54	0.159	0.366	0.160	0.367	0.161	0.367
- 55–59	0.168	0.374	0.169	0.374	0.158	0.365
- 60–64	0.152	0.359	0.153	0.360	0.130	0.336
- 65–69	0.091	0.287	0.090	0.286	0.059	0.235
Levels of Education:						
- none	0.004	0.061	0.004	0.061	0.004	0.061
- primary	0.291	0.454	0.289	0.453	0.332	0.471
- secondary	0.476	0.499	0.477	0.499	0.517	0.500
- tertiary	0.229	0.420	0.231	0.421	0.147	0.354
Number of children by age category:						
- 0–5	0.174	0.467	0.175	0.468	0.190	0.485
- 6–10	0.190	0.467	0.189	0.466	0.193	0.466
- 11–15	0.171	0.433	0.171	0.434	0.171	0.432
Employee (vs. self-employed/professional)	0.778	0.415	0.780	0.414	–	–
Years of experience	14.920	15.425	15.211	15.429	15.221	14.988
Years of tenure	–	–	13.600	11.298	11.275	10.302
Temporary job (vs. permanent)	–	–	0.117	0.322	0.143	0.350
Part-time job (vs. full-time)	–	–	0.186	0.389	0.223	0.416
Occupation (1 digit):						
- legislator, senior officials, and managers	0.025	0.157	0.025	0.157	0.010	0.101
- professionals	0.150	0.357	0.151	0.358	0.062	0.242
- technicians and associate professionals	0.178	0.382	0.179	0.383	0.166	0.372
- clerks	0.120	0.325	0.121	0.326	0.152	0.359
- service workers and shop and market sales workers	0.191	0.393	0.189	0.392	0.192	0.394
- skilled agricultural, fishery, craft and related trades workers	0.148	0.355	0.147	0.354	0.156	0.363
- plant and machine operators and assemblers	0.082	0.275	0.082	0.275	0.133	0.339
- elementary occupations	0.106	0.307	0.105	0.306	0.129	0.335
Industry (1 digit):						
- agriculture, forestry and fishing	0.042	0.200	0.041	0.199	–	–
- manufacturing	0.198	0.399	0.199	0.399	0.324	0.468
- construction	0.060	0.238	0.060	0.238	0.067	0.250
- wholesales and retail trade	0.137	0.344	0.137	0.344	0.162	0.368
- hotels and restaurants	0.063	0.243	0.061	0.239	0.076	0.265
- transport and storage	0.048	0.215	0.048	0.215	0.078	0.268
- communication	0.024	0.152	0.024	0.153	0.035	0.184
- financial intermediation	0.027	0.162	0.027	0.163	0.041	0.199
- real estate, renting, and business activities	0.110	0.313	0.111	0.314	0.118	0.322
- public administration and defence	0.051	0.220	0.052	0.221	–	–
- education, health, and social work	0.166	0.373	0.167	0.373	–	–
- other community, social, personal service activities	0.073	0.260	0.072	0.259	0.099	0.299
Plant size (n. workers):						
- less than 10	–	–	0.299	0.458	0.365	0.481
- 11–15	–	–	0.079	0.270	0.103	0.304
- 16–19	–	–	0.031	0.173	0.038	0.190
- 20–49	–	–	0.120	0.325	0.138	0.345
- 50–249	–	–	0.167	0.373	0.178	0.383
- more than 250	–	–	0.098	0.298	0.107	0.310
- missing	–	–	0.205	0.404	0.070	0.256
Share of female workers	0.444	0.285	0.445	0.285	0.407	0.286
Remote_index	50.683	10.618	50.772	10.627	51.463	10.694
Positive_pop × 1000	0.154	0.304	0.163	0.311	0.171	0.319
N. obs.	132,055		121,744		67,368	

Sample 1 for job loss includes people who are employed or have lost their job in the reference month. Sample 2 for working hours keep only individuals employed in the reference week. Sample 3 for CIG and remote working selects employees excluding those working in sectors which could not benefit from the wage guarantee fund (CIG): agriculture, forestry and fishery, public administration, defence, education, human health and social work activities, extra-territorial organisations and bodies

In our estimations we add also fixed effects of time and province of residence

## Estimation results

### Main results

In this section, we present the main results from Eq. (1) for our four outcomes of interest. According to the strategy proposed by Angrist and Pischke (2009) and Leamer (1983) to confirm the credibility of observational studies, for each outcome we report the results obtained by four different specifications. In fact, Tables 4–7 report in Column 1 the specification without any control; Column 2 includes province and time fixed effects, together with the weekly number of Covid-19 positive cases over total resident population at the province level (*z*_*p*_); Column 3 controls for individual characteristics only (*x*_*i*_), whereas Column 4 is the complete specification with individual controls, province and time fixed effects as well as the weekly rate of contagion (*x*_*i*_ and *z*_*p*_). Although our discussion is limited to the most saturated model (Column 4), results are pretty robust across specifications and confirm the stability of our estimation strategy. Complete results are reported in the Online Appendix E, Tables E.1–E.4.

**Table 4:** DDD - Job loss

	(1)	(2)	(3)	(4)
*δ*_3_ - Diff-in-Diff (ATT)	0.007***	0.007***	0.007***	0.007***
	[0.002]	[0.001]	[0.001]	[0.001]
*δ*_7_ - Diff-in-Diff-in-Diff, F vs M (gATT)	0.006**	0.006**	0.007**	0.007**
	[0.003]	[0.003]	[0.003]	[0.003]
Outcome variable means in pre-lockdown period:				
Treated M	0.005			
Control M	0.004			
Treated F	0.008			
Control F	0.006			
N	132,055	132,055	132,055	132,055
*R*^2^	0.004	0.007	0.014	0.018

Statistical significance: *0.1, **0.05, and ***0.01. Cluster-robust S.E. are reported in brackets. Complete results are reported in Appendix E, Table E.1

Model (1) includes no controls. Model (2) includes province and time fixed effects as well as the lagged weekly rate of contagion at the province level. Model (3) controls for the full set of individual and job characteristics, as described in Subsection 5.2. Model (4) is our preferred baseline model and controls for the full set of individual and job characteristics, for province and time fixed effects and for the lagged weekly rate of contagion at the province level

The treated group includes workers in non-essential sectors, whilst the control group represents workers in essential sectors

We focus on the impact of the lockdown implementation on gender inequalities as expressed by the coefficient *δ*_7_, that represents the gendered average treatment effect on the treated (gATT). We also consider the impact of lockdown per se that affected jointly male and female workers employed in non essential sectors *δ*_3_, that represents the average treatment effect on the treated (ATT), regardless of gender. However, as explained in Subsection 4.1, our DDD identification strategy allows to identify gATT (*δ*_7_) consistently, whereas the point estimates of ATT (*δ*_3_) might be biased and need to be interpreted with caution.

The evidence we get on the four outcomes is mixed. On one hand, we do observe that the lockdown apparently had an impact per se on all the outcomes for workers employed in non essential sectors compared to those employed in essential ones. On the other hand, we find a gendered impact of the lockdown among the treated group in terms of job loss as in Bluedorn et al. (2021) and in terms of the access to CIG, whereas no difference was detected as far as working hours and remote working are concerned.

More in detail, the probability of job loss became 0.7 p.p. higher among female workers compared to their male counterparts in non-essential treated sectors (Table 4, column 4, *δ*_7_), and this impact adds to the significant and positive lockdown effect eventually found for all treated workers (*δ*_3_) with respect to workers in essential sectors. If we look at the pre-lockdown probability of job loss for females in non-essential sectors, which was equal to 0.8%, we can see that the size of the gATT is comparable in absolute value. Women in non essential sectors represented the most fragile category in terms of job losses due to the lockdown.^25^

In terms of working hours, as Table 5 shows, *δ*_7_ is never statistically different from zero across specifications: this means that the drop in the intensive margin due to the lockdown was similar between female and male workers in the treated group and no additional penalty was detected for female workers.

**Table 5:** DDD—working hours

	(1)	(2)	(3)	(4)
*δ*_3_ - Diff-in-Diff (ATT)	−4.699***	−4.708***	−4.639***	−4.658***
	[0.405]	[0.398]	[0.384]	[0.380]
*δ*_7_ - Diff-in-Diff-in-Diff, F vs M (gATT)	0.141	0.031	0.099	-0.006
	[0.587]	[0.568]	[0.547]	[0.529]
Outcome variable means in pre-lockdown period:				
Treated M	39.061			
Control M	38.485			
Treated F	32.133			
Control F	30.201			
N	121,744	121,744	121,744	121,744
*R*^2^	0.072	0.145	0.212	0.284

Statistical significance: *0.1, **0.05, and ***0.01. Cluster-robust S.E. are reported in brackets. Complete results are reported in Appendix E, Table E.1

Model (1) includes no controls. Model (2) includes province and time fixed effects as well as the lagged weekly rate of contagion at the province level. Model (3) controls for the full set of individual and job characteristics, as described in Subsection 5.2. Model (4) is our preferred baseline model and controls for the full set of individual and job characteristics, for province and time fixed effects and for the lagged weekly rate of contagion at the province level

The treated group includes workers in non-essential sectors, whilst the control group represents workers in essential sectors

The probability of receiving the CIG benefit instead was 3.6 p.p. higher for female treated workers compared to male ones (Table 6),^26^ although the lockdown per se had already increased the likelyhood of benefitting from this protection measure for workers employed in non essential sectors (*δ*_3_) in comparison to the essential ones. The size of the gATT (3.6 p.p.) is sizeable when compared to a pre-lockdown average equal to 4% among female treated workers.

**Table 6:** DDD—Cig

	(1)	(2)	(3)	(4)
*δ*_3_ - Diff-in-Diff (ATT)	0.069***	0.069***	0.069***	0.069***
	[0.006]	[0.006]	[0.006]	[0.005]
*δ*_7_ - Diff-in-Diff-in-Diff, F vs M (gATT)	0.034***	0.036***	0.034***	0.036***
	[0.011]	[0.011]	[0.011]	[0.011]
Outcome variable means in pre-lockdown period:				
Treated M	0.009			
Control M	0.002			
Treated F	0.004			
Control F	0.001			
N	67,368	67,368	67,368	67,368
*R*^2^	0.042	0.115	0.072	0.141

*0.1, **0.05, and ***0.01 level of statistical significance. Cluster-robust S.E. are reported in brackets. Complete results are reported in Appendix E, Table E.3

Model (1) includes no controls. Model (2) includes province and time fixed effects as well as the lagged weekly rate of contagion at the province level. Model (3) controls for the full set of individual and job characteristics, as described in Subsection 5.2. Model (4) is our preferred baseline model and controls for the full set of individual and job characteristics, for province and time fixed effects and for the lagged weekly rate of contagion at the province level

The treated group includes workers in non-essential sectors, whilst the control group represents workers in essential sectors

When looking at remote working (Table 7), we do not observe any gendered impact in the increased probability to work from home among treated workers (*δ*_7_), although the lockdown per se decreased such probability for workers in non essential sectors compared to those employed in essential ones, regardless of gender (*δ*_3_).

**Table 7:** DDD—remote working

	(1)	(2)	(3)	(4)
*δ*_3_ - Diff-in-Diff (ATT)	−0.054***	−0.054***	−0.051***	−0.051***
	[0.015]	[0.015]	[0.015]	[0.015]
*δ*_7_ - Diff-in-Diff-in-Diff, F vs M (gATT)	0.008	0.008	0.005	0.005
	[0.021]	[0.020]	[0.021]	[0.021]
Outcome variable means in pre-lockdown period:				
Treated M	0.012			
Control M	0.025			
Treated F	0.011			
Control F	0.023			
N	67,368	67,368	67,368	67,368
*R*^2^	0.028	0.066	0.226	0.241

Statistical significance: *0.1, **0.05, and ***0.01. Cluster-robust S.E. are reported in brackets. Complete results are reported in Appendix E, Table E.4

Model (1) includes no controls. Model (2) includes province and time fixed effects as well as the lagged weekly rate of contagion at the province level. Model (3) controls for the full set of individual and job characteristics, as described in Subsection 5.2. Model (4) is our preferred baseline model and controls for the full set of individual and job characteristics, for province and time fixed effects and for the lagged weekly rate of contagion at the province level

The treated group includes workers in non-essential sectors, whilst the control group represents workers in essential sectors

### Validity tests

In Section 4.2 we discussed the assumptions under which we can credibly identify the causal impact of the lockdown due to the Covid-19 emergency on our labour market outcomes. The parallel trends assumption states that, in the absence of the lockdown, the labour market outcomes—distinguished by gender—of treated individuals employed in non-essential economic sectors and those of untreated individuals employed in essential economic sectors would have followed similar trends. We can therefore check whether male and female workers belonging to the treated and to the control group, respectively, were following parallel trends before the lockdown started. Furthermore, in the DDD model we also need to check whether female and male treated workers were following parallel trends before the lockdown started.

As in Autor (2003), we include in Eq. (1) a further set of dummy variables related to the leads of lockdown implementation from 3 to 10 relative weeks (from 1 to 2 relative months for job loss) and the lags from 11 to 41 weeks (from 3 to 9 months for job loss). This model is estimated first by splitting the sample by gender and then in the full sample across gender. In other words, for working hours, CIG, and remote working we first add *sp*_*i*,3_; ... ; *sp*_*i*,41_ indicators for males and females, separately, whilst for job loss we include *mp*_*i*,3_; ... ; *mp*_*i*,9_ indicators. Finally, to test the parallel trend across gender we add interaction terms such as *fsp*_*i*,3_; ... ; *fsp*_*i*,41_ (*fmp*_*i*,1_; ... ; *fmp*_*i*,9_ for job loss) for disentangling the impact on female and male workers. We then test whether such indicators, which would point to groups (treated vs. control) and gender (male vs. female among treated and control group, respectively) differences in the pre-treatment period, are jointly nill.

**Fig. 6:**
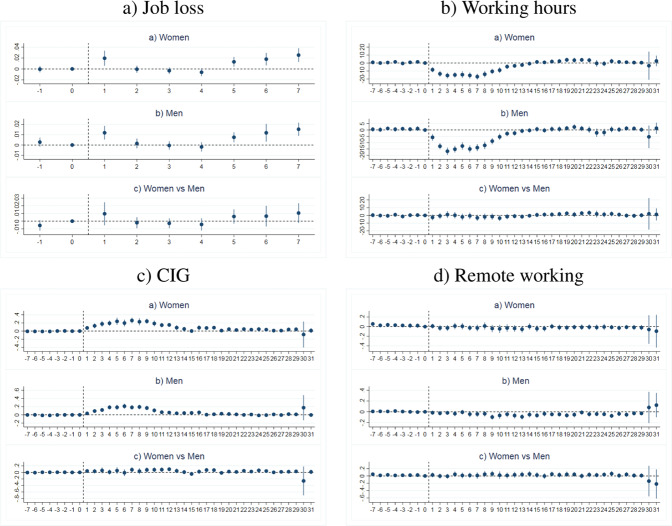
Tests for the parallel trend assumption. For each outcomes, we test parallel trends assumption in pre-lockdown period (all leads on the left-side of the vertical line) as follows: (**a**) between treated workers (non essential sectors) and control group (essential sectors) in the women subsample; (**b**) between treated workers (non essential sectors) and control group (essential sectors) in the men subsample; (**c**) between female and male treated workers (non essential sectors) in the full sample. The complete set of results is reported in Tables C.1, C.2, C.3, and C.4 in the Online Appendix C

Figure 6 shows the estimated coefficients for the time indicators for the pre-treatment and post-treatment period: the coefficients of the leads follow a flat trend that is almost stable in the period before the start of lockdown, both by gender and across gender. The complete set of results is reported in Tables C.1, C.2, C.3, and C.4 in the Appendix C. The coefficients of the leads are not jointly different from zero in any specification, except when we test remote working in the women subsample. Here the joint test on the coefficients of the pre-treatment dummies is significant at 10% (*p* value: 0.075). However, from (Fig. 6, d) we can observe that the trend before the lockdown implementation is very closed to the horizontal axis and also that, where it moves away, it does so with positive value in opposite direction to that estimated during the lockdown period. Thus, results on this outcome variable should be interpreted with caution.

A further assumption (no anticipation) would fail if individuals were able to anticipate the policy intervention and decided to close their activities or had quit their job before the actual lockdown implementation. The direction of the eventual bias could go in either way. To check whether anticipation might be an issue in our setting, we removed all individuals interviewed before the lockdown implementation, from 6th to 10th week, and re-estimated the model on this reduced sample. Results are provided in Table 8. The point estimates are very much in line with those reported in Tables 4–7 and our baseline results are confirmed, often with higher statistical significance for job loss and larger magnitude for all outcomes except remote working.

**Table 8:** Testing for the no anticipation assumption

	Job Loss	Working Hours	Cig	Remote Working
*δ*_3_ - Diff-in-Diff (ATT)	0.007***	−4.641***	0.072***	−0.056***
	[0.002]	[0.449]	[0.006]	[0.015]
*δ*_7_ - Diff-in-Diff-in-Diff, F vs M (gATT)	0.009***	−0.129	0.037***	0.001
	[0.003]	[0.612]	[0.012]	[0.022]
Outcome variables means in pre-lockdown period:				
Treated M	0.005	39.061	0.009	0.012
Control M	0.004	38.485	0.002	0.025
Treated F	0.008	32.133	0.004	0.011
Control F	0.006	30.201	0.001	0.023
N obs	119,308	109,283	60,479	60,479
*R*^2^	0.019	0.274	0.139	0.254

Statistical significance: *0.1, **0.05, and ***0.01. Cluster-robust S.E. are reported in brackets

All the models control for the full set of individual and job characteristics, as described in Subsection 5.2, for province and time fixed effects and for the lagged weekly rate of contagion at the province level. The full set of estimation results are available from the authors upon request

The treated group includes workers in non-essential sectors, whilst the control group represents workers in essential sectors

### Heterogeneity of the treatment

In this Section we further extend our baseline models by allowing the effects of the lockdown to be heterogeneous over time. Given that the vast majority of the treated sectors were able to resume their activities in May 2020 as explained in Section 3, the assumption of constant treatment effects can be removed in order to model their dynamic over time (e.g. Athey & Imbens, 2018; Callaway & Sant’Anna, 2021; de Chaisemartin & D’Haultfœuille, 2020; Sun & Abraham, 2021; Goodman-Bacon, 2021). We hence augment the previous models in Eq. (1) by distinguishing the lockdown *C* into two different treatments: the strict lockdown dummy *L*, that is equal to 1 for the period of strict closure from the 11th to the 21st week, and 0 otherwise; the post-lockdown dummy *R*, that is equal to 1 for the reopening period from the 22nd to the 39th week, and 0 otherwise.

Equation (1) then becomes:

2 $$\begin{array}{lll}{y}_{i,p,t}\,=\,{\delta }_{0}+{\delta }_{1}{S}_{i,t}+{\delta }_{2}{L}_{i,p,t}+{\delta }_{3}{R}_{i,p,t}+{\delta }_{4}{S}_{i,t}\times {L}_{i,p,t}+{\delta }_{5}{S}_{i,t}\times {R}_{i,p,t}+{\delta }_{6}fe{m}_{i}\\ \qquad\qquad+\,{\delta }_{7}{S}_{i,t}\times fe{m}_{i}+{\delta }_{8}{L}_{i,p,t}\times fe{m}_{i}+{\delta }_{9}{R}_{i,p,t}\times fe{m}_{i}\\ \qquad\qquad+\,{\delta }_{10}{S}_{i,t}\times {L}_{i,p,t}\times fe{m}_{i}+{\delta }_{11}{S}_{i,t}\times {R}_{i,p,t}\times fe{m}_{i}+{x}_{i,t}^{{\prime} }{\gamma }_{1}+{z}_{p,t}^{{\prime} }{\gamma }_{2}+{\varepsilon }_{i,p,t}.\end{array}$$

where we have two gATTs for each outcome: *δ*_10_ refers to the strict lockdown, whereas *δ*_11_ refers to the period when restrictions started to relax. Parameters are again estimated by Ordinary Least Squares (OLS) with robust standard errors clustered by sectors. This analysis regarding the heterogeneity of treatment over time is crucial to understand, firstly, whether the gATT (*δ*_7_ in Eq. (1)) remained stable over time or instead was an avarage combination of two different effects (*δ*_10_ and *δ*_11_), and, then, whether it was persistent or reabsorbed immediately.

Results are reported in Table 9. Concerning the job loss outcome, we find that the gender difference previously observed in Table 4 is not detected during the strict lockdown but rather in the reopening period, when women employed in treated sector experienced a significant increase in their job loss probability by 0.8 p.p. compared to their male counterparts, an effect that in absolute value is exactly equal to the pre-lockdown average probability of job loss for treated women (0.8%). When looking at both working hours and remote working, this augmented specification confirms results from the previous DDD model where no significant gender differences were detected among treated workers. On the other hand, the probability of benefiting from the CIG adoption was constantly larger for treated women compared to treated men, with a gap equal to 4.3 p.p. during the lockdown, and to 3.2 p.p. in the reopening phase. For both periods, the size of the impact was quite relevant compared to the mean outcome value for female treated workers in the pre-lockdown period (4%).

**Table 9:** Heterogeneous treatment: lockdown and reopening

	Job Loss	Working Hours	Cig	Remote Working
*δ*_4_ - Diff-in-Diff (ATT) of Lockdown	0.002	−12.536***	0.155***	−0.051***
	[0.001]	[0.965]	[0.012]	[0.017]
*δ*_5_ - Diff-in-Diff (ATT) of Reopening	0.010***	−0.626*	0.025***	−0.051***
	[0.002]	[0.341]	[0.004]	[0.014]
*δ*_10_ - DDD, F vs M, of Lock. (gATT)	0.005	−1,379	0.043**	0.004
	[0.003]	[1.258]	[0.022]	[0.024]
*δ*_11_ - DDD, F vs M, of Reop. (gATT)	0.008**	0.796	0.032***	0.006
	[0.004]	[0.526]	[0.009]	[0.019]
Outcome variables means in pre-lockdown period:				
Treated M	0.005	39.061	0.009	0.012
Control M	0.004	38.485	0.002	0.025
Treated F	0.008	32.133	0.004	0.011
Control F	0.006	30.201	0.001	0.023
N. obs	132,055	121,744	67,368	67,368
*R*^2^	0.018	0.309	0.152	0.240

Statistical significance: *0.1, **0.05, and ***0.01. Cluster-robust S.E. are reported in brackets.

All the models control for the full set of individual and job characteristics, as described in Subsection 5.2, for province and time fixed effects and for the lagged weekly rate of contagion at the province level. The full set of estimation results are available from the authors upon request

The treated group includes workers in non-essential sectors, whilst the control group represents workers in essential sectors

Finally, in the Online Appendix D we also test the robustness of these results by estimating the “interaction-weighted” estimator (IW) for dynamic effects using the method proposed by Sun and Abraham (2021) in order to disentangle true heterogenous effects from contaminations of other periods.

### Robustness checks

In this section we briefly mention a battery of robustness checks to test the sensitivity of our results. Detailed estimation results are reported in the Online Appendix D.

First, we estimate jointly the DDD both with the kernel propensity-score matching (PSM) model as in Heckman et al. (1998, 1997) and Blundell and Costa Dias (2009) and with the inverse probability treatment weighting (IPTW) as in Stuart et al. (2014) and Austin and Stuart (2015).^27^ In Table D.1 and D.2 we show the estimation results of our DDD setting with the kernel PSM and with the IPTW, respectively. Point estimates are very much in line with the ones reported in Subsection 4.1. Second, we examined the robustness of our findings by limiting the time horizon of the analysis. The sample is cut at the 21st week, which marks the end of the strict lockdown, before the beginning of the reopening of non essential sectors. Results reported in Table D.3 confirm those obtained for the lockdown period when considering the heterogeneity of the treatment in Subsection 6.3, Table 9.

Lastly, Table D.4 shows the estimation of heterogeneous treatment effects using the IW estimator proposed by Sun and Abraham (2021). All the main results are confirmed also with this alternative methodology.

## Conclusions

We evaluated the gendered impact of the nationwide lockdown imposed by the Italian government between March and May 2020 due to Covid-19 emergency, and how persistent it was over time. By using Labour Force Survey (LFS) data on the first three quarters of 2020, we investigated four main outcomes: job loss, hours worked per week, wage guarantee fund (CIG), and remote working. We took advantage of the exact timing of the lockdown implementation and distinguished workers employed in essential (control group) and non-essential economic sectors (treated group) in order to estimate the casual impact of such policy intervention within a Triple Difference-in-Differences (DDD) design to analyse whether gender inequalities emerged, and get a consistent estimate of their size in terms of gendered average treatment effect on the treated (gATT).

In the treated group, the lockdown somehow enlarged pre-existent gender inequalities, but we failed to detect a homogeneous pattern across the different outcomes. The probability of job loss got 0.7 p.p. higher among female workers compared to their male counterparts in treated sectors and this difference was detected during the reopening period rather than in the strict lockdown phase. The probability of receiving CIG benefit was 3.6 p.p. higher for female treated workers compared to their male counterparts, despite the fact that men were more likely to benefit from this measure before the lockdown due to theit higher incidence in manufacturing sectors where the use of CIG was traditionally allowed. The higher incidence of CIG among female treated workers was detected both during the lockdown and the reopening phase. No significant gender differences emerged either in terms of working hours or in terms of remote working among the treated group, at least in the medium-term.

We can conclude that in Italy the gendered impact due to the lockdown implementation, when significant, was mainly detected with regard to the extensive margin of labour market participation, and to the reopening period. Social protection measures such as the CIG extension helped mitigating the dramatic consequences of the lockdown, and these effects were particularly significant for female workers employed in non–essential sectors. In the absence of these interventions, women would have probably suffered a much worse impact.

However, a deeper investigation of the potential heterogeneity of the results in terms of gender gap across workers with different care responsibilities (e.g. with or without children) could be object of future research, particularly in order to investigate if any post-Covid-19 policy intervention should be designed to stimulate the employability and allow for a better work-life balance of mothers.

## Supplementary information

Supplementary Information

## Footnotes

1 According to Istat, the difference between male and female employment rates of the age group 15–64 was 24.1 in 2004 and 18.1 in 2020. See http://dati.istat.it.

2 Labour income does not appear among our outcomes for two reasons. Firstly, the survey question asks about last month earnings whereas other essential information, such as working hours, is not available with the same timing. Existing evidence has shown that the lockdown implementation had a large impact on the gender gap in terms of working hours (e.g. Alon et al., 2022; Meekes et al., 2022) and controlling for such information would be necessary to avoid spurious relations. Secondly, we cannot exclude that the self-reported information on labour earnings include also the wage guarantee fund (CIG) received by workers (see Section 3.2); in this case, we may not be able to distinguish it from the salary.

3 The 14 provinces outside Lombardia were Alessandria, Asti, Modena, Novara, Padua, Parma, Pesaro and Urbino, Piacenza, Reggio-Emilia, Rimini, Treviso, Venice, Verbano-Cusio-Ossola, and Vercelli.

4 The 2007 Ateco classification is the national version of the European nomenclature Nace Rev. 2 adopted by Istat in 2008.

5 For a detailed description see Biasi (2020) and Istat (2021).

6 As further explained in Subsection 5.1, three out of four outcomes (hours worked per week, wage guarantee fund (CIG), and smart/remote working) are reported on a weekly basis, whereas the probability of job loss is reported on a monthly basis.

7 As discussed above in Subsection 3.1, the policy intervention started in week 11 nationwide and was anticipated to week 9 in the provinces of Padua and Lodi. For the job loss outcome, available only on a monthly basis, we consider that the lockdown started in March.

8 See Subsection 6.1 for additional details.

9 In Subsection 6.1 the Tables 7 we report also estimations without the individual controls variables and results remain robust.

10 Time fixed effects are expressed in terms of “relative” weeks from the beginning of the lockdown for estimations of worked hours, CIG, and remote working, whereas they refer to months for job loss estimation.

11 As pointed out by Heckman et al. (1997), the inclusion of an observation sample that belongs to the region of non-overlapping support may generate serious evaluation biases.

12 Elaborations by the authors have been conducted at the Istat ADELE (Laboratorio per l’Analisi dei Dati ELEmentari) laboratory in Ancona in compliance with the norms on statistical confidentiality and personal data protection. No sample weights were employed in our estimates.

13 The size of sample_2_ is lower than sample_1_ one because the first one collects all workers at reference time, whilst the latter also includes individuals who have lost their job in the current month.

14 Starting from *s**a**m**p**l**e*_2_, we kept only employees and among employees we further excluded those working in the following sectors which could not benefit from the wage guarantee fund (CIG): agriculture, forestry and fishery, public administration, defence, education, human health and social work activities, extra-territorial organisations and bodies.

15 Sectors were excluded based on the possibility to switch jobs into remote working. Neither agriculture, forestry and fishery, nor the health sector could do it, whilst public administration and education had to do it.

16 The availability of the latter set of information is conditional on the individual employment status, i.e. they are absent for those who were unemployed at the time of the interview.

17 Information on the years of tenure and on the type of contract are available only for individuals who were employed at the time of the interview, *s**a**m**p**l**e*_2 and *s**a**m**p**l**e*_3.

18 The ATECO 2007 classification is the Italian equivalent of the NACE Rev. 2 classification of economic activities by Eurostat.

19 We also add a further category in which to group employees with missing information on the size of the firm they work in.

20 This share is computed using the *s**a**m**p**l**e*_2_ of all employees as the average share of women over the weeks before the lockdown implementation by 3-digit sector.

21 The survey consider about 16,000 workers employed in around 800 occupations, according to the 5-digit CP2011 classification (the Italian equivalent of the ISCO-08 ILO’s classification). The ICP survey is based on the US Occupational Information Network (O*Net) run by the Bureau of Labour Statistics. It investigates the characteristics of the occupations through a particularly rich and articulated questionnaire structured in seven sections (knowledge, skills, attitudes, generalized work activities, values, work styles and working conditions) and it describes all the professions existing in the Italian labour market (e.g. those operating in private companies, those present in public institutions and state-owned enterprises, and those carried out by the self-employed and regulated professionals). Each answer to these questions are on a 0–5 scale based on how frequent or important is the activity, work style or working conditions (where 5 means very frequent or very important). A score on a 0–100 scale (from less to more intense) is then calculated for each 5-digit occupation following this standardization formula:

$$X=\frac{Y-min}{max-min}* 100,$$

where Y is the original answer (from 1 to 5) and *min* and *max* are the minimum and maximum value reported for that occupation. Each value for each occupation is than standardized over the about twenty answers received from workers in that occupation. The index, therefore, has no cardinal interpretation.

22 Note that this *remote*_*index* is similar to the offshorability index by Autor and Dorn (2013), the face-to-face and on-site job indexes by Firpo et al. (2011), and the measure of safe jobs recently developed by Boeri et al. (2020), even though these indexes are all based on O*Net questions.

23 The first series comes from the website https://github.com/DavideMagno/ItalianCovidDat, the second one is retrieved from the ISTAT website.

24 For the province of Padua and Lodi the lockdown had already begun in the 9th week, as discussed in section 3. So, we transform the calendar weeks in relative weeks in base on the actual start of the lockdown. The week before the shutdown has been set to 0.

25 Interestingly, no significant gender difference in the probability of job loss was observed before the lockdown either for the treated (*δ*_5_) or for the control group (*δ*_4_) in Table E.1 of the Online Appendix E.

26 As shown in Table E.3 in the Online Appendix E, this result is independent on the fact that before the lockdown men were usually more likely to benefit from this measure (*δ*_5_). No significant gender differences were instead detected in the control group (*δ*_6_) due to the policy implementation.

27 We used the STATA command diff for the estimation of Triple Diff-in-Diff (DDD) model reweighted with the kernel density PSM model (command psmacht2) as well as with IPTW (command mlogit) on the subsamples based on common support.
