# Supplementary material for: The impact of Covid-19 lockdown on the gender gap in the Italian labour market
Source: Rev Econ Househ. 2023 Jun 1:1–33. Online ahead of print. doi: 10.1007/s11150-023-09659-5 (PMC10234238; doi:10.1007/s11150-023-09659-5)
Supplement: Supplementary file 1 — Supplementary Information [file 11150_2023_9659_MOESM1_ESM.pdf]

Online Appendix

A Methodology

As in Olden and Møen (2022), we start from Equation 1 in Subsection 4.1 and simplify the Triple Difference-in-Differences (DDD) specification[28] into a basic setup with all the dummy variables as follows:

$$y_{i,p,t} = \delta_0 + \delta_1 S_{i,t} + \delta_2 C_{i,p,t} + \delta_3 S_{i,t} \times C_{i,p,t} + \delta_4 fem_i + \delta_5 S_{i,t} \times fem_i + \\ + \delta_6 C_{i,p,t} \times fem_i + \delta_7 S_{i,t} \times C_{i,p,t} \times fem_i + \varepsilon_{i,p,t}. \tag{A.1}$$

Now, the conditional mean function of Equation A.1 is $E[y_{i,p,t} \mid S, fem, C]$, which can take on eight values. Under standard OLS assumptions and an addictive effects, we can use $E[\varepsilon_{i,p,t} \mid S, fem, C] = 0$ to show the eight expected values as in A.2.

$$\begin{aligned}
&E[y_{i,p,t} \mid S = 0, fem = 0, C = 0] = \delta_0 \\
&E[y_{i,p,t} \mid S = 1, fem = 0, C = 0] = \delta_0 + \delta_1 \\
&E[y_{i,p,t} \mid S = 0, fem = 0, C = 1] = \delta_0 + \delta_2 \\
&E[y_{i,p,t} \mid S = 0, fem = 1, C = 0] = \delta_0 + \delta_4 \\
&E[y_{i,p,t} \mid S = 1, fem = 1, C = 0] = \delta_0 + \delta_1 + \delta_4 + \delta_5 \\
&E[y_{i,p,t} \mid S = 1, fem = 0, C = 1] = \delta_0 + \delta_1 + \delta_2 + \delta_3 \\
&E[y_{i,p,t} \mid S = 0, fem = 1, C = 1] = \delta_0 + \delta_2 + \delta_4 + \delta_6 \\
&E[y_{i,p,t} \mid S = 1, fem = 1, C = 1] = \delta_0 + \delta_1 + \delta_2 + \delta_3 + \delta_4 + \delta_5 + \delta_6 + \delta_7.
\end{aligned} \tag{A.2}$$

From the Equation A.1 we can solve for the δ as follows:

[28] See Olden and Møen (2022) for a review of the literature concerning the DDD methodology.

$$
\begin{aligned}
\delta_0 =& E[y_{i,p,t} \mid S=0, fem=0, C=0] \\
\delta_1 =& E[y_{i,p,t} \mid S=1, fem=0, C=0] - E[y_{i,p,t} \mid S=0, fem=0, C=0] \\
\delta_2 =& E[y_{i,p,t} \mid S=0, fem=0, C=1] - E[y_{i,p,t} \mid S=0, fem=0, C=0] \\
\delta_4 =& E[y_{i,p,t} \mid S=0, fem=1, C=0] - E[y_{i,p,t} \mid S=0, fem=0, C=0] \\
\delta_5 =& E[y_{i,p,t} \mid S=1, fem=1, C=0] + E[y_{i,p,t} \mid S=0, fem=0, C=0] + \\
& - E[y_{i,p,t} \mid S=1, fem=0, C=0] - E[y_{i,p,t} \mid S=0, fem=1, C=0] \\
\delta_3 =& E[y_{i,p,t} \mid S=1, fem=0, C=1] + E[y_{i,p,t} \mid S=0, fem=0, C=0] + \\
& - E[y_{i,p,t} \mid S=1, fem=0, C=0] - E[y_{i,p,t} \mid S=0, fem=0, C=1] \\
\delta_6 =& E[y_{i,p,t} \mid S=0, fem=1, C=1] + E[y_{i,p,t} \mid S=0, fem=0, C=0] + \\
& - E[y_{i,p,t} \mid S=0, fem=1, C=0] - E[y_{i,p,t} \mid S=0, fem=0, C=1] \\
\delta_7 =& [(E[y_{i,p,t} \mid S=1, fem=1, C=1] - E[y_{i,p,t} \mid S=1, fem=1, C=0]) + \\
& - (E[y_{i,p,t} \mid S=1, fem=0, C=1] - E[y_{i,p,t} \mid S=1, fem=0, C=0])] + \\
& - [(E[y_{i,p,t} \mid S=0, fem=1, C=1] - E[y_{i,p,t} \mid S=0, fem=1, C=0]) + \\
& - (E[y_{i,p,t} \mid S=0, fem=0, C=1] - E[y_{i,p}, t \mid S=0, fem=0, C=0])].
\end{aligned} \tag{A.3}
$$

By rearranging the expression for δ_7 in terms of expected values, we obtain the triple difference estimator as in the following Equation A.4:

$$
\begin{aligned}
\hat{\delta_7} = & [(\bar{Y}_{S=1,fem=1,C=1} - \bar{Y}_{S=1,fem=1,C=0}) - (\bar{Y}_{S=0,fem=1,C=1} - \bar{Y}_{S=0,fem=1,C=0})] + \\
& -[(\bar{Y}_{S=1,fem=0,C=1} - \bar{Y}_{S=1,fem=0,C=0}) - (\bar{Y}_{S=0,fem=0,C=1} - \bar{Y}_{S=0,fem=0,C=0})].
\end{aligned} \tag{A.4}
$$

B Further descriptive statistics

Table B.1: Samples - Frequencies by groups and periods

	Sample 1 - Job loss			Sample 2 - Weekly working hours			Sample 3 - Cig			Sample 3 - Remote working		
	Before/After	Lockdown	Post lockdown	Before/After	Lockdown	Post lockdown	Before/After	Lockdown	Post lockdown	pre/post	Lockdown	Post lockdown
All												
Before, Treated	10,971	0	0	9,074	0	0	6,432	0	0	6,432	0	0
Before, Control	21,183	0	0	17,718	0	0	8,615	0	0	8,615	0	0
After, Treated	33,623	14,095	19,528	31,627	10,639	20,988	22,257	7,508	14,749	22,257	10,493	11,764
After, Control	66,278	28,573	37,705	63,325	22,075	41,250	30,064	10,346	19,718	30,064	14,254	15,810
Total	132,055	42,668	57,233	121,744	32,714	62,238	67,368	17,854	34,467	67,368	24,747	27,574
Males												
Before, Treated	6,965	0	0	5,749	0	0	3,956	0	0	3,956	0	0
Before, Control	10,887	0	0	9,112	0	0	4,890	0	0	4,890	0	0
After, Treated	21,476	9,012	12,464	20,205	6,795	13,410	13,955	4,703	9,252	13,955	6,561	7,394
After, Control	33,814	14,542	19,272	32,182	11,128	21,054	16,905	5,854	11,051	16,905	8,049	8,856
Total	73,142	23,554	31,736	67,248	17,923	34,464	39,706	10,557	20,303	39,706	14,610	16,250
Females												
Before, Treated	4,006	0	0	3,325	0	0	2,476	0	0	2,476	0	0
Before, Control	10,296	0	0	8,606	0	0	3,725	0	0	3,725	0	0
After, Treated	12,147	5,083	7,064	11,422	3,844	7,578	8,302	2,805	5,497	8,302	3,932	4,370
After, Control	32,464	14,031	18,433	31,143	10,947	20,196	13,159	4,492	8,667	13,159	6,205	6,954
Total	58,913	19,114	25,497	54,496	14,791	27,774	27,662	7,297	14,164	27,662	10,137	11,324

Notes: Sample 1 for job loss includes people who are employed or have lost their job in the reference month. Sample 2 for working hours keep only individuals employed in the reference week. Sample 3 for CIG and remote working selects employees excluding those working in sectors which could not benefit from the wage guarantee fund (CIG): agriculture, forestry and fishery, public administration, defence, education, human health and social work activities, extra-territorial organisations and bodies.
Column Before/After refers to periods of the lockdown implementation: period Before counts from 3th to 10th week (and January and February for Job loss), whereas period After includes weeks from the 11th to the 39th one (from March to September for Job loss). Column Lockdown refers only the period of lockdown, in particular from the 11th to the 21st one (for Job loss are months from March to May). Column Post lockdown represents the period of activities reopening after the lockdown, such as from the 22nd to the 39th week (from June to September for Job loss).
Sample 3 differs in columns Lockdown and Post lockdown for CIG and Remote working because of the nature of available data. As explained in Subsection 5.1, the information of CIG regards the reference week whereas that of remote working refers to a period that includes the reference week and the previous three.
The treated group includes workers in non-essential sectors, whilst the control group represents workers in essential sectors.

Table B.2: Outcomes - Means by groups and periods

	Sample 1 Job loss			Sample 2 Working Hours			Sample 3 Cig			Remote working		
	Before/After	Lockdown	Post lockdown	Before/After	Lockdown	Post lockdown	Before/After	Lockdown	Post lockdown	pre/post	Lockdown	Post lockdown
All												
Before, Treated	0.006	-	-	36,522	-	-	0.007	-	-	0.012	-	-
Before, Control	0.005	-	-	34,461	-	-	0.002	-	-	0.024	-	-
After, Treated	0.013	0.010	0.014	27,300	16,124	32,965	0.143	0.281	0.072	0.070	0.089	0.054
After, Control	0.002	0.004	0.001	29,894	27,349	31,256	0.056	0.106	0.030	0.135	0.156	0.117
Total	0.006	0.006	0.006	30,379	23,699	31,833	0.073	0.179	0.048	0.088	0.127	0.090
Males												
Before, Treated	0.005	-	-	39,061	-	-	0.009	-	-	0.012	-	-
Before, Control	0.004	-	-	38,485	-	-	0.002	-	-	0.025	-	-
After, Treated	0.010	0.008	0.011	29,489	17,974	35,324	0.131	0.270	0.061	0.061	0.078	0.046
After, Control	0.002	0.005	0.001	33,612	29,920	35,563	0.056	0.108	0.028	0.128	0.144	0.114
Total	0.005	0.006	0.005	33,499	25,391	35,470	0.071	0.180	0.043	0.080	0.114	0.083
Females												
Before, Treated	0.008	-	-	32,133	-	-	0.004	-	-	0.011	-	-
Before, Control	0.006	-	-	30,201	-	-	0.001	-	-	0.023	-	-
After, Treated	0.018	0.013	0.020	23,426	12,852	28,789	0.161	0.301	0.090	0.086	0.108	0.067
After, Control	0.002	0.004	0.001	26,053	24,736	26,767	0.056	0.102	0.031	0.144	0.171	0.121
Total	0.006	0.006	0.006	26,528	21,648	27,319	0.075	0.179	0.054	0.099	0.146	0.100

Notes: Sample 1 for job loss includes people who are employed or have lost their job in the reference month. Sample 2 for working hours keep only individuals employed in the reference week. Sample 3 for CIG and remote working selects employees excluding those working in sectors which could not benefit from the wage guarantee fund (CIG): agriculture, forestry and fishery, public administration, defence, education, human health and social work activities, extra-territorial organisations and bodies.
Column Before/After refers to periods of the lockdown implementation: period Before counts from 3th to 10th week (and January and February for Job loss), whereas period After includes weeks from the 11th to the 39th one (from March to September for Job loss). Column Lockdown refers only the period of lockdown, in particular from the 11th to the 21st one (for Job loss are months from March to May). Column Post lockdown represents the period of activities reopening after the lockdown, such as from the 22nd to the 39th week (from June to September for Job loss).
The treated group includes workers in non-essential sectors, whilst the control group represents workers in essential sectors.

C Validity tests

Table C.1: Parallel trend test for DDD model of Equation 1 - Job loss

	only women		only men		women vs men	
	β	(s.e.)	β	(s.e.)	β	(s.e.)
δ_1 - Before, Treated vs Control	0.00771***	(0.00280)	0.00200	(0.00163)	0.00198	(0.00163)
δ_5 - Before, Treated vs Control, F vs M					0.00573*	(0.00317)
mp1	-0.000537	(0.00297)	0.00281	(0.00223)	0.000527	(0.00236)
mp3	0.0196***	(0.00703)	0.0119***	(0.00336)	0.00662*	(0.00368)
mp4	-0.000538	(0.00327)	0.00152	(0.00241)	0.00158	(0.00241)
mp5	-0.00326	(0.00304)	-0.000432	(0.00211)	-0.000417	(0.00210)
mp6	-0.00611	(0.00373)	-0.00184	(0.00229)	-0.00184	(0.00229)
mp7	0.0130***	(0.00453)	0.00751***	(0.00250)	0.00950***	(0.00252)
mp8	0.0179***	(0.00583)	0.0119***	(0.00437)	0.0142***	(0.00450)
mp9	0.0253***	(0.00641)	0.0152***	(0.00329)	0.0184***	(0.00348)
fmp1					-0.00564	(0.00356)
fmp3					0.00954	(0.00764)
fmp4					-0.00212	(0.00362)
fmp5					-0.00284	(0.00326)
fmp6					-0.00427	(0.00407)
fmp7					0.00599	(0.00476)
fmp8					0.00647	(0.00685)
fmp9					0.0105	(0.00654)
N	58913		73142		132055	
R^2	0.024		0.018		0.022	
Joint F test of pre-treatment dummies	0.033		1.594		2.503	
p-value	0.857		0.207		0.114	

Notes: *** Significant at 1%; ** significant at 5%; * significant at 10%. Cluster-robust S.E. are reported in brackets.
The treated group includes workers in non-essential sectors, whilst the control group represents workers in essential sectors. We control for the full set of individual and job characteristics, as described in Subsection 5.2, for province and time fixed effects and for the lagged weekly rate of contagion at the province level. The full set of estimation results are available from the authors upon request.

Table C.2: Parallel trend test for DDD model of Equation 1 - Working hours

	only women		only men		women vs men	
	β	(s.e.)	β	(s.e.)	β	(s.e.)
δ_1 - Before, Treated vs Control	-1.220	(0.958)	-0.683	(0.751)	-0.683	(0.751)
δ_5 - Before, Treated vs Control, F vs M					-0.537	(1.109)
sp3	0.804	(1.291)	0.482	(0.899)	0.482	(0.900)
sp4	-0.0100	(1.190)	0.203	(0.913)	0.203	(0.913)

Continued on next page

Table C.2 – continued from previous page

	only women		only men		women vs men	
	β	(s.e.)	β	(s.e.)	β	(s.e.)
sp5	0.582	(1.262)	1.143	(0.830)	1.143	(0.831)
sp6	1.384	(1.123)	0.495	(0.909)	0.495	(0.909)
sp7	-0.492	(1.179)	0.917	(0.894)	0.917	(0.894)
sp8	0.857	(1.196)	0.591	(0.906)	0.591	(0.906)
sp9	1.426	(0.939)	1.028	(0.970)	1.028	(0.970)
sp11	-8.423***	(1.617)	-5.835***	(1.362)	-5.835***	(1.362)
sp12	-13.56***	(1.840)	-12.80***	(1.259)	-12.80***	(1.259)
sp13	-15.70***	(1.799)	-16.78***	(1.483)	-16.78***	(1.483)
sp14	-14.96***	(1.735)	-15.14***	(1.369)	-15.14***	(1.369)
sp15	-14.64***	(2.149)	-12.60***	(1.509)	-12.60***	(1.510)
sp16	-15.58***	(1.935)	-14.97***	(1.424)	-14.97***	(1.424)
sp17	-17.07***	(1.808)	-13.93***	(1.392)	-13.93***	(1.392)
sp18	-14.08***	(2.025)	-12.13***	(1.506)	-12.13***	(1.506)
sp19	-10.48***	(1.775)	-8.815***	(1.285)	-8.815***	(1.286)
sp20	-8.921***	(1.799)	-5.404***	(1.248)	-5.404***	(1.249)
sp21	-4.537***	(1.484)	-2.872***	(1.110)	-2.872***	(1.110)
sp22	-3.547***	(1.293)	-2.501**	(1.035)	-2.501**	(1.035)
sp23	-2.443*	(1.291)	-0.769	(0.954)	-0.769	(0.955)
sp24	-0.758	(1.159)	-0.309	(0.897)	-0.309	(0.897)
sp25	1.293	(1.309)	0.635	(0.976)	0.635	(0.976)
sp26	0.792	(1.132)	-0.298	(0.928)	-0.298	(0.928)
sp27	1.842	(1.468)	0.691	(1.088)	0.691	(1.089)
sp28	2.341*	(1.322)	0.675	(1.094)	0.675	(1.094)
sp29	3.950***	(1.498)	1.373	(0.974)	1.373	(0.974)
sp30	3.544**	(1.603)	2.359**	(1.038)	2.359**	(1.038)
sp31	3.929***	(1.327)	1.145	(1.058)	1.145	(1.059)
sp32	3.556**	(1.463)	0.232	(1.284)	0.232	(1.284)
sp33	-0.392	(2.127)	-2.223	(1.681)	-2.223	(1.681)
sp34	-0.743	(1.652)	-1.973	(1.505)	-1.973	(1.505)
sp35	2.376	(1.475)	0.452	(1.189)	0.452	(1.189)
sp36	1.354	(1.213)	0.318	(0.972)	0.318	(0.973)
sp37	0.995	(1.208)	1.259	(0.937)	1.259	(0.938)
sp38	0.579	(1.194)	1.101	(0.962)	1.101	(0.962)
sp39	0.423	(1.237)	0.234	(0.886)	0.234	(0.886)
sp40	-3.410	(9.016)	-5.405	(4.525)	-5.405	(4.526)
sp41	2.618	(3.374)	1.354	(2.256)	1.354	(2.257)
fsp3					0.322	(1.429)
fsp4					-0.213	(1.367)
fsp5					-0.561	(1.395)
fsp6					0.889	(1.319)
fsp7					-1.409	(1.435)
fsp8					0.265	(1.316)

Continued on next page

Table C.2 – continued from previous page

	only women		only men		women vs men	
	β	(s.e.)	β	(s.e.)	β	(s.e.)
fsp9					0.398	(1.285)
fsp11					-2.588	(1.893)
fsp12					-0.755	(1.984)
fsp13					1.076	(2.281)
fsp14					0.177	(2.083)
fsp15					-2.032	(2.229)
fsp16					-0.609	(2.162)
fsp17					-3.133	(1.968)
fsp18					-1.948	(2.158)
fsp19					-1.669	(2.106)
fsp20					-3.517*	(1.936)
fsp21					-1.665	(1.763)
fsp22					-1.046	(1.534)
fsp23					-1.674	(1.482)
fsp24					-0.449	(1.325)
fsp25					0.658	(1.466)
fsp26					1.090	(1.386)
fsp27					1.150	(1.541)
fsp28					1.666	(1.448)
fsp29					2.577*	(1.552)
fsp30					1.184	(1.708)
fsp31					2.784*	(1.477)
fsp32					3.324**	(1.629)
fsp33					1.831	(2.247)
fsp34					1.230	(1.791)
fsp35					1.925	(1.627)
fsp36					1.036	(1.371)
fsp37					-0.264	(1.461)
fsp38					-0.522	(1.460)
fsp39					0.189	(1.433)
fsp40					1.995	(10.380)
fsp41					1.264	(3.996)
N. obs	54496		67248		121744	
R^2	0.313		0.272		0.319	
Joint F test of pre-treatment dummies	1.650		0.556		0.840	
p−value	0.119		0.792		0.554	

Notes: *** Significant at 1%; ** significant at 5%; * significant at 10%. Cluster-robust S.E. are reported in brackets. The treated group includes workers in non-essential sectors, whilst the control group represents workers in essential sectors. We control for the full set of individual and job characteristics, as described in Subsection 5.2, for province and time fixed effects and for the lagged weekly rate of contagion at the province level. The full set of estimation results are available from the authors upon request.

Table C.3: Parallel trend test for DDD model of Equation 1 - Cig

	only women		only men		women vs men	
	β	(s.e.)	β	(s.e.)	β	(s.e.)
δ_1 - Before, Treated vs Control	-0.0180*	(0.00931)	0.00150	(0.00771)	0.00150	(0.00772)
δ_5 - Before, Treated vs Control, F vs M					-0.0195*	(0.0108)
sp3	-0.00694	(0.00972)	-0.00108	(0.00998)	-0.00108	(0.00998)
sp4	-0.00863	(0.00929)	0.000965	(0.00851)	0.000965	(0.00852)
sp5	-0.00983	(0.00847)	-0.0123	(0.00825)	-0.0123	(0.00825)
sp6	-0.00983	(0.00978)	-0.0132	(0.00845)	-0.0132	(0.00846)
sp7	0.00115	(0.00760)	-0.00222	(0.0115)	-0.00222	(0.0115)
sp8	0.00291	(0.00790)	-0.000109	(0.00846)	-0.000109	(0.00846)
sp9	-0.00232	(0.00674)	-0.000110	(0.0110)	-0.000110	(0.0110)
sp11	0.0770***	(0.0229)	0.0344**	(0.0160)	0.0344**	(0.0161)
sp12	0.129***	(0.0311)	0.0940***	(0.0238)	0.0940***	(0.0238)
sp13	0.177***	(0.0359)	0.119***	(0.0262)	0.119***	(0.0262)
sp14	0.193***	(0.0335)	0.184***	(0.0256)	0.184***	(0.0256)
sp15	0.239***	(0.0459)	0.184***	(0.0312)	0.184***	(0.0312)
sp16	0.198***	(0.0418)	0.211***	(0.0312)	0.211***	(0.0312)
sp17	0.261***	(0.0349)	0.182***	(0.0266)	0.182***	(0.0266)
sp18	0.229***	(0.0447)	0.192***	(0.0291)	0.192***	(0.0291)
sp19	0.242***	(0.0317)	0.168***	(0.0278)	0.168***	(0.0278)
sp20	0.188***	(0.0365)	0.109***	(0.0261)	0.109***	(0.0261)
sp21	0.146***	(0.0287)	0.0649***	(0.0245)	0.0649***	(0.0245)
sp22	0.151***	(0.0233)	0.0565***	(0.0215)	0.0565***	(0.0215)
sp23	0.0835***	(0.0315)	0.0372	(0.0244)	0.0372	(0.0244)
sp24	0.0509*	(0.0265)	0.0436**	(0.0189)	0.0436**	(0.0189)
sp25	0.00155	(0.0241)	0.0483**	(0.0218)	0.0483**	(0.0218)
sp26	0.0822***	(0.0302)	0.0603***	(0.0191)	0.0603***	(0.0191)
sp27	0.0751***	(0.0244)	0.00436	(0.0153)	0.00436	(0.0153)
sp28	0.0831***	(0.0186)	0.0148	(0.0121)	0.0148	(0.0121)
sp29	0.0121	(0.0203)	0.0287*	(0.0158)	0.0287*	(0.0159)
sp30	0.0462**	(0.0223)	0.0215	(0.0140)	0.0215	(0.0140)
sp31	0.0268	(0.0167)	0.0122	(0.0144)	0.0122	(0.0144)
sp32	0.0482***	(0.0177)	-0.000215	(0.0101)	-0.000215	(0.0101)
sp33	0.0346*	(0.0202)	0.00950	(0.0120)	0.00950	(0.0120)
sp34	0.0473***	(0.0141)	-0.0131	(0.0125)	-0.0131	(0.0126)
sp35	0.0348**	(0.0140)	-0.00654	(0.0101)	-0.00654	(0.0101)
sp36	0.0111	(0.0118)	0.0114	(0.0129)	0.0114	(0.0129)
sp37	0.0118	(0.0141)	-0.00432	(0.0112)	-0.00432	(0.0112)
sp38	0.0399***	(0.0133)	0.0188	(0.0123)	0.0188	(0.0123)
sp39	0.0433**	(0.0188)	0.0123	(0.0133)	0.0123	(0.0133)
sp40	-0.0858	(0.163)	0.176	(0.156)	0.176	(0.156)
sp41	0.0118	(0.0300)	-0.00288	(0.0215)	-0.00288	(0.0215)
fsp3					-0.00586	(0.0119)

Continued on next page

Table C.3 – continued from previous page

	only women		only men		women vs men	
	β	(s.e.)	β	(s.e.)	β	(s.e.)
fsp4					-0.00959	(0.0112)
fsp5					0.00247	(0.0110)
fsp6					0.00338	(0.0123)
fsp7					0.00338	(0.0125)
fsp8					0.00302	(0.0119)
fsp9					-0.00221	(0.0122)
fsp11					0.0426	(0.0261)
fsp12					0.0345	(0.0351)
fsp13					0.0582	(0.0463)
fsp14					0.00896	(0.0400)
fsp15					0.0550	(0.0527)
fsp16					-0.0127	(0.0522)
fsp17					0.0789*	(0.0413)
fsp18					0.0365	(0.0474)
fsp19					0.0739*	(0.0385)
fsp20					0.0788**	(0.0380)
fsp21					0.0813**	(0.0358)
fsp22					0.0944***	(0.0317)
fsp23					0.0462	(0.0357)
fsp24					0.00730	(0.0333)
fsp25					-0.0468	(0.0324)
fsp26					0.0220	(0.0343)
fsp27					0.0708**	(0.0288)
fsp28					0.0683***	(0.0230)
fsp29					-0.0166	(0.0230)
fsp30					0.0247	(0.0275)
fsp31					0.0146	(0.0212)
fsp32					0.0484**	(0.0194)
fsp33					0.0251	(0.0221)
fsp34					0.0604***	(0.0190)
fsp35					0.0414**	(0.0164)
fsp36					-0.000273	(0.0162)
fsp37					0.0161	(0.0178)
fsp38					0.0211	(0.0171)
fsp39					0.0310	(0.0200)
fsp40					-0.261	(0.225)
fsp41					0.0147	(0.0353)
N. obs	27662		39706		67368	
R^2	0.174		0.152		0.161	
Joint F test of pre-treatment dummies	0.737		1.794		0.454	
p−value	0.641		0.866		0.867	

Continued on next page

Table C.3 – continued from previous page

	only women		only men		women vs men	
	β	(s.e.)	β	(s.e.)	β	(s.e.)

Notes: *** Significant at 1%; ** significant at 5%; * significant at 10%. Cluster-robust S.E. are reported in brackets. The treated group includes workers in non-essential sectors, whilst the control group represents workers in essential sectors. We control for the full set of individual and job characteristics, as described in Subsection 5.2, for province and time fixed effects and for the lagged weekly rate of contagion at the province level. The full set of estimation results are available from the authors upon request.

Table C.4: Parallel trend test for DDD model of Equation 1 - Remote working

	only women		only men		women vs men	
	β	(s.e.)	β	(s.e.)	β	(s.e.)
δ_1 - Before, Treated vs Control	0.0110	(0.0227)	0.0305**	(0.0151)	0.0305**	(0.0152)
δ_5 - Before, Treated vs Control, F vs M					-0.0195	(0.0250)
sp3	0.0568***	(0.0206)	0.00772	(0.0150)	0.00772	(0.0150)
sp4	0.0257	(0.0189)	0.00937	(0.0138)	0.00937	(0.0138)
sp5	0.0373*	(0.0219)	0.00691	(0.0140)	0.00691	(0.0140)
sp6	0.0301*	(0.0169)	0.0149	(0.0153)	0.0149	(0.0153)
sp7	0.0243	(0.0196)	0.00523	(0.0140)	0.00523	(0.0140)
sp8	0.0211	(0.0255)	-0.000410	(0.0175)	-0.000410	(0.0175)
sp9	0.0208	(0.0236)	-0.00509	(0.0172)	-0.00509	(0.0172)
sp11	0.0105	(0.0253)	-0.0175	(0.0202)	-0.0175	(0.0202)
sp12	-0.0299	(0.0315)	-0.0274	(0.0218)	-0.0274	(0.0218)
sp13	-0.0279	(0.0315)	-0.0198	(0.0194)	-0.0198	(0.0195)
sp14	0.0121	(0.0342)	-0.0346	(0.0240)	-0.0346	(0.0240)
sp15	0.00798	(0.0344)	-0.00671	(0.0234)	-0.00671	(0.0234)
sp16	-0.0258	(0.0291)	-0.0428	(0.0282)	-0.0428	(0.0283)
sp17	-0.0287	(0.0350)	-0.0411	(0.0257)	-0.0411	(0.0257)
sp18	0.0131	(0.0383)	-0.0364	(0.0257)	-0.0364	(0.0257)
sp19	-0.0393	(0.0390)	-0.0985***	(0.0244)	-0.0985***	(0.0244)
sp20	-0.0468	(0.0438)	-0.0679**	(0.0318)	-0.0679**	(0.0318)
sp21	-0.0300	(0.0398)	-0.0449	(0.0287)	-0.0449	(0.0287)
sp22	-0.0374	(0.0353)	-0.0699**	(0.0281)	-0.0699**	(0.0281)
sp23	-0.0542	(0.0344)	-0.0941***	(0.0241)	-0.0941***	(0.0241)
sp24	0.00241	(0.0331)	-0.0526**	(0.0268)	-0.0526*	(0.0268)
sp25	-0.0428	(0.0332)	-0.0676***	(0.0231)	-0.0676***	(0.0231)
sp26	-0.0395	(0.0299)	-0.0369	(0.0246)	-0.0369	(0.0246)
sp27	0.00471	(0.0272)	-0.0470**	(0.0204)	-0.0470**	(0.0204)
sp28	-0.0172	(0.0296)	-0.0473**	(0.0202)	-0.0473**	(0.0202)
sp29	-0.0220	(0.0295)	-0.0667***	(0.0237)	-0.0667***	(0.0237)
sp30	-0.00996	(0.0258)	-0.0540**	(0.0247)	-0.0540**	(0.0248)
sp31	-0.0127	(0.0296)	-0.0120	(0.0208)	-0.0120	(0.0208)

Continued on next page

Table C.4 – continued from previous page

	only women		only men		women vs men	
	β	(s.e.)	β	(s.e.)	β	(s.e.)
sp32	-0.00905	(0.0285)	-0.0441*	(0.0231)	-0.0441*	(0.0231)
sp33	-0.0160	(0.0302)	-0.0410**	(0.0205)	-0.0410**	(0.0205)
sp34	-0.00632	(0.0329)	-0.0444**	(0.0204)	-0.0444**	(0.0204)
sp35	-0.0130	(0.0251)	-0.0761***	(0.0207)	-0.0761***	(0.0207)
sp36	-0.0277	(0.0260)	-0.0378*	(0.0214)	-0.0378*	(0.0214)
sp37	-0.0113	(0.0245)	-0.0537**	(0.0226)	-0.0537**	(0.0226)
sp38	-0.0158	(0.0280)	-0.0286	(0.0212)	-0.0286	(0.0212)
sp39	-0.0197	(0.0271)	-0.0303	(0.0192)	-0.0303	(0.0192)
sp40	-0.0594	(0.151)	0.0813	(0.148)	0.0813	(0.148)
sp41	-0.0950	(0.173)	0.124	(0.115)	0.124	(0.115)
fsp3					0.0491**	(0.0238)
fsp4					0.0164	(0.0223)
fsp5					0.0304	(0.0257)
fsp6					0.0152	(0.0226)
fsp7					0.0191	(0.0233)
fsp8					0.0215	(0.0280)
fsp9					0.0259	(0.0286)
fsp11					0.0280	(0.0339)
fsp12					-0.002	(0.0342)
fsp13					-0.008	(0.0334)
fsp14					0.0467	(0.0384)
fsp15					0.0147	(0.0402)
fsp16					0.0170	(0.0372)
fsp17					0.0123	(0.0424)
fsp18					0.0495	(0.0428)
fsp19					0.0592	(0.0405)
fsp20					0.0212	(0.0525)
fsp21					0.0150	(0.0412)
fsp22					0.0325	(0.0414)
fsp23					0.0399	(0.0384)
fsp24					0.0550	(0.0397)
fsp25					0.0248	(0.0373)
fsp26					-0.003	(0.0382)
fsp27					0.0517	(0.0334)
fsp28					0.0301	(0.0347)
fsp29					0.0447	(0.0378)
fsp30					0.0440	(0.0325)
fsp31					-0.000648	(0.0351)
fsp32					0.0351	(0.0362)
fsp33					0.0250	(0.0327)
fsp34					0.0381	(0.0336)
fsp35					0.063**	(0.0319)

Continued on next page

Table C.4 – continued from previous page

	only women		only men		women vs men	
	β	(s.e.)	β	(s.e.)	β	(s.e.)
fsp36					0.0102	(0.0319)
fsp37					0.0424	(0.0329)
fsp38					0.0128	(0.0334)
fsp39					0.0107	(0.0306)
fsp40					-0.141	(0.213)
fsp41					-0.219	(0.203)
N. obs	27662		39706		67368	
R^2	0.234		0.257		0.247	
Joint F test of pre-treatment dummies	1.862		0.486		0.929	
p−value	0.075		0.845		0.484	

Notes: *** Significant at 1%; ** significant at 5%; * significant at 10%. Cluster-robust S.E. are reported in brackets. The treated group includes workers in non-essential sectors, whilst the control group represents workers in essential sectors. We control for the full set of individual and job characteristics, as described in Subsection 5.2, for province and time fixed effects and for the lagged weekly rate of contagion at the province level. The full set of estimation results are available from the authors upon request.

D Robustness analysis

Table D.1: Triple Diff-in-Diff (DDD) estimations with Kernel PSM

	Job Loss		Working Hours		Cig		Remote Working	
	b	s.e.	b	s.e.	b	s.e.	b	s.e.
Before								
Control (F)	0.016		35.223		0.100		-0.173	
Control (M)	0.015		38.645		0.099		-0.170	
Treated (F)	0.017		35.001		0.101		-0.175	
Treated (M)	0.016		37.969		0.105		-0.174	
Diff (T-C)	0.000	0.002	0.454	0.503	-0.004	0.003	0.002	0.010
After								
Control (F)	0.012		31.006		0.161		-0.096	
Control (M)	0.013		33.369		0.153		-0.103	
Treated (F)	0.026		25.993		0.255		-0.107	
Treated (M)	0.020		28.336		0.224		-0.125	
Diff (T-C)	0.006***	0.002	0.020	0.603	0.023*	0.013	0.010	0.007
DDD	0.006**	0.003	-0.434	0.607	0.028**	0.013	0.008	0.013
N	129,479		121,733		67,367		67,367	
r2	0.02		0.19		0.07		0.19	

Notes: *** Significant at 1%; ** significant at 5%; * significant at 10%. Cluster-robust S.E. are reported. For estimations we use the Stata commands `psmatch2 kernel` option for the PSM model and then `diff` for the triple DDD model.

The treated group includes workers in non-essential sectors, whilst the control group represents workers in essential sectors. We control for the full set of individual and job characteristics, as described in Subsection 5.2, for province and time fixed effects and for the lagged weekly rate of contagion at the province level. The full set of estimation results are available from the authors upon request.

Table D.2: Triple Diff-in-Diff (DDD) estimations with IPT weights

	Job Loss		Working Hours		Cig		Remote Working	
	b	s.e.	b	s.e.	b	s.e.	b	s.e.
Before								
Control (F)	0.017		33.738		0.106		-0.189	
Control (M)	0.016		37.802		0.105		-0.174	
Treated (F)	0.019		33.786		0.108		-0.185	
Treated (M)	0.017		36.930		0.112		-0.182	
Diff (T-C)	0.000	0.002	0.921***	0.326	-0.005*	0.003	0.012***	0.004
After								
Control (F)	0.014		29.540		0.162		-0.103	
Control (M)	0.015		32.482		0.160		-0.113	
Treated (F)	0.029		24.778		0.262		-0.117	
Treated (M)	0.022		27.296		0.231		-0.133	
Diff (T-C)	0.008***	0.002	0.423	0.266	0.029***	0.006	0.006	0.004
DDD	0.007***	0.002	-0.497	0.419	0.034***	0.006	-0.006	0.006
N	129,479		121,733		67,367		67,367	
R^2	0.02		0.20		0.07		0.19	

Notes: *** Significant at 1%; ** significant at 5%; * significant at 10%. Cluster-robust S.E. are reported. For estimations we use the Stata commands `mlogit` for calculating the IPT weights and then `diff` for the triple DDD model.

The treated group includes workers in non-essential sectors, whilst the control group represents workers in essential sectors. We control for the full set of individual and job characteristics, as described in Subsection 5.2, for province and time fixed effects and for the lagged weekly rate of contagion at the province level. The full set of estimation results are available from the authors upon request.

Table D.3: Estimated effects of lockdown after limiting the sample at the 21st week

	Job Loss	Working Hours	Cig	Remote Working
δ_0 Before, Control	0.017***	30.396***	0.240***	0.010
	[0.006]	[2.712]	[0.031]	[0.044]
δ_1 - Before, Treated vs Control	-0.000	0.828	-0.005	0.031***
	[0.001]	[0.522]	[0.007]	[0.011]
δ_2 - Control, After vs Before	0.006***	-1.021*	-0.015	0.104***
	[0.002]	[0.557]	[0.009]	[0.018]
δ_3 - Diff-in-Diff (ATET)	0.002	-12.161***	0.149***	-0.052***
	[0.002]	[0.945]	[0.012]	[0.017]
δ_4 - Female	0.002	-3.790***	0.008	-0.005
	[0.001]	[0.407]	[0.006]	[0.017]
δ_5 - Before, Treated vs Control, F vs M	-0.002	0.378	-0.003	0.006
	[0.002]	[0.577]	[0.008]	[0.015]
δ_6 - Control, After vs Before, F vs M	-0.003*	2.860***	-0.002	0.023
	[0.002]	[0.691]	[0.012]	[0.026]
δ_7 - Diff-in-Diff-in-Diff, F vs M	0.005	-1,180	0.032	0.003
	[0.003]	[1.236]	[0.021]	[0.024]
N obs.	68,280	62,725	34,701	39,794
R^2	0.018	0.338	0.181	0.229

Notes: *** Significant at 1%; ** significant at 5%; * significant at 10%. Cluster-robust S.E. are reported in brackets.

The treated group includes workers in non-essential sectors, whilst the control group represents workers in essential sectors. We control for the full set of individual and job characteristics, as described in Subsection 5.2, for province and time fixed effects and for the lagged weekly rate of contagion at the province level. The full set of estimation results are available from the authors upon request.

Table D.4: Heterogeneous treatment: lockdown and reopening - IW estimations

	Job Loss	Working Hours	Cig	Remote Working
δ_0 - Before, Control	0.010**	35.926***	0.108***	0.024
	[0.005]	[2.507]	[0.021]	[0.042]
δ_1 - Before, Treated vs Control	0.003**	-0.228	-0.004	0.035***
	[0.001]	[0.362]	[0.005]	[0.012]
δ_2 - Control, Lock-down vs Before	0.001	-2.597***	-0.010	0.090***
	[0.001]	[0.673]	[0.011]	[0.017]
δ_3 - Control, Reopening vs Before	-0.005***	-1.528**	0.114***	0.070*
	[0.001]	[0.597]	[0.010]	[0.039]
δ_4 - Diff-in-Diff (ATET) of Lockdown	0.002	-12.536***	0.155***	-0.051***
	[0.001]	[0.965]	[0.012]	[0.017]
δ_5 - Diff-in-Diff (ATET) of Reopening	0.010***	-0.626*	0.025***	-0.051***
	[0.002]	[0.341]	[0.004]	[0.014]
δ_6 - Female	0.001	-2.926***	0.004	-0.003
	[0.001]	[0.322]	[0.005]	[0.018]
δ_7 - Before, Treat. vs Contr., F vs M	-0.000	-0.366	-0.014**	0.005
	[0.002]	[0.475]	[0.006]	[0.016]
δ_8 - Control, Lock. vs Bef., F vs M	-0.003*	2.968***	-0.004	0.023
	[0.001]	[0.745]	[0.014]	[0.026]
δ_9 - Control, Reop. vs Bef., F vs M	-0.002	-0.707*	0.005	0.007
	[0.001]	[0.380]	[0.005]	[0.019]
δ_{10} - DDD, F vs M, of Lock.	0.005	-1,379	0.043**	0.004
	[0.003]	[1.258]	[0.022]	[0.024]
δ_{11} - DDD, F vs M, of Reop.	0.008**	0.796	0.032***	0.006
	[0.004]	[0.526]	[0.009]	[0.019]
N. obs	132,055	121,744	67,368	67,368
R^2	0.018	0.309	0.152	0.240

Notes: * 0.1, ** 0.05, and *** 0.01 levels of statitical significance. Cluster-robust S.E. are reported in brackets.

The treated group includes workers in non-essential sectors, whilst the control group represents workers in essential sectors. All the models control for the full set of individual and job characteristics, as described in Subsection 5.2, for province and time fixed effects and for the lagged weekly rate of contagion at the province level. The full set of estimation results are available from the authors upon request.

E Full set of estimation results of the benchmark models

Table E.1: Full set of results of the estimates reported in Table 4 - Job loss

	Job loss			
	(1)	(2)	(3)	(4)
δ_0 - Before, Control	0.004***	0.001	0.011**	0.010**
	[0.001]	[0.001]	[0.004]	[0.005]
δ_1 - Before, Treated vs Control	0.001	0.002	0.003**	0.003**
	[0.001]	[0.001]	[0.001]	[0.001]
δ_2 - Control, After vs Before	-0.002**	0.004**	-0.002**	0.004**
	[0.001]	[0.002]	[0.001]	[0.002]
δ_3 - Diff-in-Diff (ATET)	0.007***	0.007***	0.007***	0.007***
	[0.002]	[0.001]	[0.001]	[0.001]
δ_4 - Female	0.002	0.002	0.001	0.001
	[0.001]	[0.001]	[0.001]	[0.001]
δ_5 - Before, Treated vs Control, F vs M	0.001	0.001	-0.000	-0.000
	[0.002]	[0.002]	[0.002]	[0.002]
δ_6 - Control, After vs Before, F vs M	-0.002	-0.002	-0.002	-0.002
	[0.001]	[0.001]	[0.001]	[0.001]
δ_7 - Diff-in-Diff-in-Diff, F vs M	0.006**	0.006**	0.007**	0.007**
	[0.003]	[0.003]	[0.003]	[0.003]
Foreign citizenship	-	-	-0.000	-0.000
			[0.001]	[0.001]
Age cohorts - Reference: 30-34				
- 20-24	-	-	0.021**	0.020**
			[0.009]	[0.009]
- 25-29	-	-	0.005**	0.005**
			[0.002]	[0.002]
- 35-39	-	-	-0.003*	-0.003*
			[0.002]	[0.002]
- 40-44	-	-	-0.002	-0.002
			[0.001]	[0.001]
- 45-49	-	-	-0.002*	-0.002
			[0.001]	[0.001]
- 50-54	-	-	-0.001	-0.001
			[0.001]	[0.001]
- 55-59	-	-	-0.000	0.000
			[0.001]	[0.001]
- 60-64	-	-	0.001	0.002
			[0.002]	[0.002]
- 65-69	-	-	0.008***	0.009***
			[0.002]	[0.002]
Levels of Educations - Reference: secondary				
- none	-	-	0.003	0.003
			[0.004]	[0.004]
- primary	-	-	0.001**	0.001***

Continued on next page

Table E.1 – continued from previous page

	Job loss			
	(1)	(2)	(3)	(4)
			[0.001]	[0.001]
- tertiary	-	-	-0.001	-0.001
			[0.001]	[0.001]
Remote_index	-	-	-0.000	-0.000
			[0.000]	[0.000]
Industry (1 digit) - Reference: communication				
- agriculture, forestry, and fishing	-	-	0.002	0.002
			[0.003]	[0.003]
- manufacturing	-	-	-0.006***	-0.006***
			[0.001]	[0.001]
- construction	-	-	-0.002	-0.002
			[0.002]	[0.002]
- wholesales and retail trade	-	-	-0.004***	-0.005***
			[0.002]	[0.002]
- hotels and resturants	-	-	0.002	0.001
			[0.005]	[0.004]
- transport and storage	-	-	0.000	0.000
			[0.001]	[0.001]
- financial intermediation	-	-	-0.001	-0.002
			[0.001]	[0.001]
- real estate, renting, and business activities	-	-	-0.002	-0.002*
			[0.001]	[0.001]
- public administration and defence	-	-	-0.001	-0.001
			[0.001]	[0.001]
- education, health, and social work	-	-	-0.002	-0.002*
			[0.001]	[0.001]
- other community, social, personal service activities	-	-	-0.001	-0.001
			[0.002]	[0.002]
Occupation (1 digit) - Reference: Clerks				
- legislator, senior officials, and managers	-	-	-0.003*	-0.002*
			[0.001]	[0.001]
- professionals	-	-	0.000	0.000
			[0.001]	[0.001]
- technicians and associate professionals	-	-	-0.001	-0.001
			[0.001]	[0.001]
- service workers and shop and market sales workers	-	-	0.001	0.001
			[0.002]	[0.002]
- skilled agriculturals, fishery, craft and related trades workers	-	-	-0.001	-0.001
			[0.002]	[0.002]
- plant and machine operators and assemblers	-	-	0.000	0.000
			[0.002]	[0.002]
- elementary occupations	-	-	0.003	0.003

Continued on next page

Table E.1 – continued from previous page

			Job loss	
	(1)	(2)	(3)	(4)
			[0.002]	[0.002]
Employee (vs. self-employed/professional)	-	-	0.005***	0.005***
			[0.001]	[0.001]
Plant size (n. workers) - Reference: less than 10				
- 11-15	-	-	-	-
- 16-19	-	-	-	-
- 20-49	-	-	-	-
- 50-249	-	-	-	-
- more than 250	-	-	-	-
- missing	-	-	-	-
Years of experience	-	-	-0.000***	-0.000***
			[0.000]	[0.000]
Years of tenure	-	-	-	-
Number of children by age category:				
- 0-5	-	-	-0.001**	-0.001**
			[0.000]	[0.000]
- 6-10	-	-	0.001	0.001
			[0.001]	[0.001]
- 11-15	-	-	0.000	0.000
			[0.000]	[0.000]
Part-time job (vs. full-time)	-	-	-	-
Temporary job (vs. permanent)	-	-	-	-
Positive_pop	-	2.919**	-	2.826**
		[1.196]		[1.181]
Female workers proportion	-	-	0.000	0.000
			[0.002]	[0.002]
N. obs 132055	132055	132055	132055	
R^2	0.004	0.007	0.014	0.018

Notes: * 0.1, ** 0.05, and *** 0.01 level of statistical significance. Cluster-robust S.E. are reported in brackets. The treated group includes workers in non-essential sectors, whilst the control group represents workers in essential sectors. Model (1) includes no controls. Model (2) includes province and time fixed effects as well as the lagged weekly rate of contagion at the province level. Model (3) controls for the full set of individual and job characteristics. Model (4) is our preferred baseline model and controls for the full set of individual and job characteristics, for province and time fixed effects and for the lagged weekly rate of contagion at the province level. The estimated coefficients of all fixed effects are not reported, they are available from the authors upon request.

Table E.2: Full set of results of the estimates reported in Table 5 - Working Hours

	Working Hours			
	(1)	(2)	(3)	(4)
δ_0 - Before, Control	38.485***	35.233***	39.228***	35.992***
	[0.459]	[0.656]	[2.522]	[2.502]
δ_1 - Before, Treated vs Control	0.577	0.449	-0.179	-0.199
	[0.595]	[0.604]	[0.362]	[0.363]
δ_2 - Control, After vs Before	-4.873***	-3.927***	-5.028***	-4.187***
	[0.309]	[0.635]	[0.298]	[0.624]
δ_3 - Diff-in-Diff (ATET)	-4.699***	-4.708***	-4.639***	-4.658***
	[0.405]	[0.398]	[0.384]	[0.380]
δ_4 - Female	-8.283***	-8.410***	-2.799***	-2.954***
	[0.740]	[0.738]	[0.320]	[0.321]
δ_5 - Before, Treated vs Control, F vs M	1,355	1,480	-0.529	-0.354
	[0.946]	[0.950]	[0.472]	[0.474]
δ_6 - Control, After vs Before, F vs M	0.724**	0.806**	0.527	0.600
	[0.355]	[0.352]	[0.371]	[0.367]
δ_7 - Diff-in-Diff-in-Diff, F vs M	0.141	0.031	0.099	-0.006
	[0.587]	[0.568]	[0.547]	[0.529]
Foreign citizenship	-	-	1.165**	0.994**
			[0.483]	[0.505]
Age cohorts - Reference: 30-34				
- 20-24	-	-	1.061*	0.212
			[0.600]	[0.562]
- 25-29	-	-	-0.180	-0.237
			[0.298]	[0.285]
- 35-39	-	-	0.069	0.071
			[0.255]	[0.249]
- 40-44	-	-	0.115	0.141
			[0.272]	[0.252]
- 45-49	-	-	0.080	0.032
			[0.287]	[0.271]
- 50-54	-	-	-0.053	-0.054
			[0.301]	[0.287]
- 55-59	-	-	-0.249	-0.245
			[0.325]	[0.313]
- 60-64	-	-	-0.935**	-0.899**
			[0.407]	[0.406]
- 65-69	-	-	-1.389**	-1.336**
			[0.568]	[0.565]
Levels of Educations - Reference: secondary				
- none	-	-	1,097	1.938***
			[0.731]	[0.616]
- primary	-	-	-0.307*	-0.265*

Continued on next page

Table E.2 – continued from previous page

	Working Hours			
	(1)	(2)	(3)	(4)
			[0.157]	[0.154]
- tertiary	-	-	1.526***	1.538***
			[0.301]	[0.300]
Remote_index	-	-	0.093**	0.098***
			[0.037]	[0.036]
Industry (1 digit) - Reference: communication				
- agriculture, forestry, and fishing	-	-	6.551***	6.325***
			[1.722]	[1.709]
- manufacturing	-	-	0.236	0.298
			[0.641]	[0.595]
- construction	-	-	-1.877***	-1.887***
			[0.721]	[0.679]
- wholesales and retail trade	-	-	0.923	1,032
			[0.795]	[0.762]
- hotels and resturants	-	-	-1,162	-1,371
			[1.169]	[1.154]
- transport and storage	-	-	-0.884	-0.735
			[0.876]	[0.840]
- financial intermediation	-	-	-0.109	-0.127
			[0.772]	[0.739]
- real estate, renting, and business activities	-	-	-0.679	-0.602
			[0.676]	[0.619]
- public administration and defence	-	-	-1.300**	-1.157**
			[0.617]	[0.578]
- education, health, and social work	-	-	-4.450***	-4.350***
			[1.033]	[0.970]
- other community, social, personal service activities	-	-	-0.227	-0.190
			[0.991]	[0.957]
Occupation (1 digit) - Reference: Clerks				
- legislator, senior officials, and managers	-	-	3.561***	3.608***
			[0.748]	[0.758]
- professionals	-	-	-3.270***	-3.130***
			[1.010]	[0.998]
- technicians and associate professionals	-	-	-0.091	-0.089
			[0.543]	[0.533]
- service workers and shop and market sales workers	-	-	3.241***	3.373***
			[1.050]	[1.033]
- skilled agriculturals, fishery, craft and related trades workers	-	-	-0.276	-0.155
			[0.862]	[0.851]
- plant and machine operators and assemblers	-	-	-0.881	-0.815
			[0.714]	[0.705]
- elementary occupations	-	-	-1,234	-1,155

Continued on next page

Table E.2 – continued from previous page

	Working Hours			
	(1)	(2)	(3)	(4)
			[0.866]	[0.843]
Employee (vs. self-employed/professional)	-	-	-3.780***	-4.115***
			[0.608]	[0.610]
Plant size (n. workers) - Reference: less than 10				
- 11-15	-	-	-0.253	0.068
			[0.236]	[0.235]
- 16-19	-	-	-0.237	0.263
			[0.319]	[0.325]
- 20-49	-	-	-0.459	-0.284
			[0.318]	[0.316]
- 50-249	-	-	-0.547	-0.375
			[0.411]	[0.424]
- more than 250	-	-	1.188**	1.377***
			[0.483]	[0.461]
- missing	-	-	-1.776***	-2.052***
			[0.348]	[0.328]
Years of experience	-	-	0.025***	0.021***
			[0.005]	[0.004]
Years of tenure	-	-	0.019*	0.018
			[0.012]	[0.012]
Number of children by age category:				
- 0-5	-	-	-1.225***	-1.210***
			[0.139]	[0.137]
- 6-10	-	-	0.104	0.119
			[0.115]	[0.113]
- 11-15	-	-	-0.160	-0.081
			[0.122]	[0.118]
Part-time job (vs. full-time)	-	-	-14.211***	-14.205***
			[0.475]	[0.469]
Temporary job (vs. permanent)	-	-	0.699**	0.584*
			[0.353]	[0.324]
Positive_pop	-	-394,766	-	-585.979**
		[261.812]		[242.415]
Female workers proportion	-	-	-2.534**	-2.412**
			[1.009]	[1.006]
N. obs	121744	121744	121744	121744
R^2	0.072	0.145	0.212	0.284

Notes: * 0.1, ** 0.05, and *** 0.01 level of statistical significance. Cluster-robust S.E. are reported in brackets.

The treated group includes workers in non-essential sectors, whilst the control group represents workers in essential sectors. Model (1) includes no controls. Model (2) includes province and time fixed effects as well as the lagged weekly rate of contagion at the province level. Model (3) controls for the full set of individual and job characteristics. Model (4) is our preferred baseline model and controls for the full set of individual and job characteristics, for province and time fixed effects and for the lagged weekly rate of contagion at the province level. The estimated coefficients of all fixed effects are not reported, they are available from the authors upon request.

Table E.3: Full set of results of the estimates reported in Table 6 - CIG

	CIG			
	(1)	(2)	(3)	(4)
δ_0 - Before, Control	0.002***	0.013*	0.095***	0.107***
	[0.001]	[0.008]	[0.022]	[0.021]
δ_1 - Before, Treated vs Control	0.007***	0.009***	-0.004	-0.004
	[0.002]	[0.002]	[0.004]	[0.005]
δ_2 - Control, After vs Before	0.053***	0.019**	0.050***	0.017**
	[0.004]	[0.008]	[0.004]	[0.008]
δ_3 - Diff-in-Diff (ATET)	0.069***	0.069***	0.069***	0.069***
	[0.006]	[0.006]	[0.006]	[0.005]
δ_4 - Female	-0.001	0.000	0.003	0.004
	[0.001]	[0.001]	[0.005]	[0.005]
δ_5 - Before, Treated vs Control, F vs M	-0.004**	-0.006***	-0.012**	-0.014**
	[0.002]	[0.002]	[0.006]	[0.006]
δ_6 - Control, After vs Before, F vs M	0.001	0.001	0.002	0.002
	[0.007]	[0.007]	[0.007]	[0.007]
δ_7 - Diff-in-Diff-in-Diff, F vs M	0.034***	0.036***	0.034***	0.036***
	[0.011]	[0.011]	[0.011]	[0.011]
Foreign citizenship	-	-	-0.023***	-0.021***
			[0.004]	[0.004]
Age cohorts - Reference: 30-34				
- 20-24	-	-	-0.023**	-0.011
			[0.010]	[0.009]
- 25-29	-	-	-0.012**	-0.013***
			[0.005]	[0.005]
- 35-39	-	-	-0.010*	-0.006
			[0.006]	[0.005]
- 40-44	-	-	0.004	0.007
			[0.006]	[0.005]
- 45-49	-	-	0.012**	0.015***
			[0.006]	[0.006]
- 50-54	-	-	0.010*	0.014***
			[0.005]	[0.005]
- 55-59	-	-	0.023***	0.025***
			[0.006]	[0.006]
- 60-64	-	-	0.028***	0.031***
			[0.007]	[0.007]
- 65-69	-	-	0.024***	0.028***
			[0.007]	[0.007]
Levels of Educations - Reference: secondary				
- none	-	-	-0.031**	-0.040***
			[0.014]	[0.013]
- primary	-	-	0.006*	0.005

Continued on next page

Table E.3 – continued from previous page

	CIG			
	(1)	(2)	(3)	(4)
			[0.003]	[0.003]
- tertiary	-	-	-0.016***	-0.016***
			[0.004]	[0.004]
Remote_index	-	-	-0.001**	-0.001***
			[0.000]	[0.000]
Industry (1 digit) - Reference: communication				
- agriculture, forestry, and fishing	-	-	-	-
- manufacturing	-	-	-0.004	-0.003
			[0.007]	[0.007]
- construction	-	-	-0.001	-0.002
			[0.009]	[0.008]
- wholesales and retail trade	-	-	-0.003	-0.002
			[0.008]	[0.008]
- hotels and resturants	-	-	0.068***	0.071***
			[0.012]	[0.012]
- transport and storage	-	-	-0.002	-0.003
			[0.009]	[0.009]
- financial intermediation	-	-	-0.036***	-0.035***
			[0.007]	[0.007]
- real estate, renting, and business activities	-	-	-0.001	-0.002
			[0.007]	[0.007]
- public administration and defence	-	-	-	-
- education, health, and social work	-	-	-	-
- other community, social, personal service activities	-	-	-0.027***	-0.026***
			[0.010]	[0.010]
Occupation (1 digit) - Reference: Clerks				
- legislator, senior officials, and managers	-	-	-0.037***	-0.041***
			[0.011]	[0.011]
- professionals	-	-	-0.026***	-0.026***
			[0.007]	[0.007]
- technicians and associate professionals	-	-	-0.006	-0.006
			[0.007]	[0.006]
- service workers and shop and market sales workers	-	-	-0.026**	-0.026**
			[0.011]	[0.011]
- skilled agriculturals, fishery, craft and related trades workers	-	-	0.005	0.006
			[0.010]	[0.010]
- plant and machine operators and assemblers	-	-	0.007	0.007
			[0.009]	[0.009]
- elementary occupations	-	-	-0.025**	-0.024**

Continued on next page

Table E.3 – continued from previous page

	CIG			
	(1)	(2)	(3)	(4)
			[0.010]	[0.010]
Employee (vs. self-employed/professional)	-	-	-	-
Plant size (n. workers) - Reference: less than 10				
- 11-15	-	-	0.002	-0.003
			[0.004]	[0.004]
- 16-19	-	-	-0.005	-0.008
			[0.005]	[0.005]
- 20-49	-	-	-0.004	-0.005
			[0.004]	[0.005]
- 50-249	-	-	-0.006	-0.007
			[0.004]	[0.004]
- more than 250	-	-	0.005	0.003
			[0.007]	[0.006]
- missing	-	-	-0.028***	-0.016***
			[0.004]	[0.004]
Years of experience	-	-	-0.001***	-0.001***
			[0.000]	[0.000]
Years of tenure	-	-	-0.001***	-0.001***
			[0.000]	[0.000]
Number of children by age category:				
- 0-5	-	-	0.001	0.001
			[0.002]	[0.002]
- 6-10	-	-	-0.002	-0.002
			[0.002]	[0.002]
- 11-15	-	-	0.006**	0.006**
			[0.003]	[0.002]
Part-time job (vs. full-time)	-	-	0.008**	0.008**
			[0.004]	[0.004]
Temporary job (vs. permanent)	-	-	-0.105***	-0.098***
			[0.009]	[0.008]
Positive_pop	-	1,822	-	3,449
		[5.268]		[5.208]
Female workers proportion	-	-	0.009	0.007
			[0.011]	[0.011]
N. obs	67368	67368	67368	67368
R^2	0.042	0.115	0.072	0.141

Notes: * 0.1, ** 0.05, and *** 0.01 level of statistical significance. Cluster-robust S.E. are reported in brackets. The treated group includes workers in non-essential sectors, whilst the control group represents workers in essential sectors. Model (1) includes no controls. Model (2) includes province and time fixed effects as well as the lagged weekly rate of contagion at the province level. Model (3) controls for the full set of individual and job characteristics. Model (4) is our preferred baseline model and controls for the full set of individual and job characteristics, for province and time fixed effects and for the lagged weekly rate of contagion at the province level. The estimated coefficients of all fixed effects are not reported, they are available from the authors upon request.

Table E.4: Full set of results of the estimates reported in Table 7 - Remote Working

	Remote Working			
	(1)	(2)	(3)	(4)
δ_0 - Before, Control	0.025***	0.114***	-0.029	0.016
	[0.004]	[0.021]	[0.043]	[0.042]
δ_1 - Before, Treated vs Control	-0.013***	-0.011***	0.036***	0.035***
	[0.004]	[0.004]	[0.012]	[0.012]
δ_2 - Control, After vs Before	0.104***	0.049***	0.101***	0.051***
	[0.017]	[0.014]	[0.017]	[0.014]
δ_3 - Diff-in-Diff (ATET)	-0.054***	-0.054***	-0.051***	-0.051***
	[0.015]	[0.015]	[0.015]	[0.015]
δ_4 - Female	-0.002	-0.006	-0.002	-0.003
	[0.004]	[0.004]	[0.017]	[0.018]
δ_5 - Before, Treated vs Control, F vs M	0.002	0.001	0.006	0.005
	[0.005]	[0.005]	[0.016]	[0.016]
δ_6 - Control, After vs Before, F vs M	0.018	0.018	0.014	0.014
	[0.022]	[0.022]	[0.022]	[0.022]
δ_7 - Diff-in-Diff-in-Diff, F vs M	0.008	0.008	0.005	0.005
	[0.021]	[0.020]	[0.021]	[0.021]
Foreign citizenship	-	-	-0.005**	-0.010***
			[0.003]	[0.003]
Age cohorts - Reference: 30-34				
- 20-24	-	-	0.023***	0.028***
			[0.008]	[0.008]
- 25-29	-	-	0.009**	0.009**
			[0.004]	[0.004]
- 35-39	-	-	0.009**	0.010**
			[0.004]	[0.004]
- 40-44	-	-	0.008*	0.008
			[0.005]	[0.005]
- 45-49	-	-	0.015***	0.014***
			[0.005]	[0.005]
- 50-54	-	-	0.010*	0.009*
			[0.005]	[0.005]
- 55-59	-	-	0.009	0.008
			[0.005]	[0.006]
- 60-64	-	-	0.006	0.005
			[0.006]	[0.006]
- 65-69	-	-	0.010	0.010
			[0.007]	[0.007]
Levels of Educations - Reference: secondary				
- none	-	-	-0.004	0.002
			[0.006]	[0.006]
- primary	-	-	-0.010***	-0.010***

Continued on next page

Table E.4 – continued from previous page

	Remote Working			
	(1)	(2)	(3)	(4)
			[0.003]	[0.003]
- tertiary	-	-	0.077***	0.074***
			[0.008]	[0.008]
Remote_index	-	-	0.004***	0.003***
			[0.001]	[0.001]
Industry (1 digit) - Reference: communication				
- agriculture, forestry, and fishing	-	-	-	-
- manufacturing	-	-	-0.209***	-0.198***
			[0.017]	[0.017]
- construction	-	-	-0.187***	-0.178***
			[0.017]	[0.018]
- wholesales and retail trade	-	-	-0.194***	-0.186***
			[0.018]	[0.018]
- hotels and resturants	-	-	-0.187***	-0.177***
			[0.020]	[0.020]
- transport and storage	-	-	-0.213***	-0.205***
			[0.018]	[0.018]
- financial intermediation	-	-	-0.083***	-0.079***
			[0.022]	[0.022]
- real estate, renting, and business activities	-	-	-0.155***	-0.149***
			[0.019]	[0.019]
- public administration and defence	-	-	-	-
- education, health, and social work	-	-	-	-
- other community, social, personal service activities	-	-	-0.182***	-0.174***
			[0.018]	[0.018]
Occupation (1 digit) - Reference: Clerks				
- legislator, senior officials, and managers	-	-	0.115***	0.108***
			[0.027]	[0.026]
- professionals	-	-	0.125***	0.122***
			[0.026]	[0.025]
- technicians and associate professionals	-	-	0.046***	0.045***
			[0.015]	[0.015]
- service workers and shop and market sales workers	-	-	0.000	0.001
			[0.022]	[0.021]
- skilled agriculturals, fishery, craft and related trades workers	-	-	-0.035**	-0.031**
			[0.015]	[0.015]
- plant and machine operators and assemblers	-	-	-0.061***	-0.056***
			[0.015]	[0.014]
- elementary occupations	-	-	-0.048***	-0.047***

Continued on next page

Table E.4 – continued from previous page

	Remote Working			
	(1)	(2)	(3)	(4)
			[0.015]	[0.015]
Employee (vs. self-employed/professional)	-	-	-	-
Plant size (n. workers) - Reference: less than 10				
- 11-15	-	-	0.006	0.006*
			[0.004]	[0.004]
- 16-19	-	-	0.014**	0.014**
			[0.006]	[0.006]
- 20-49	-	-	0.031***	0.029***
			[0.007]	[0.007]
- 50-249	-	-	0.053***	0.047***
			[0.009]	[0.008]
- more than 250	-	-	0.116***	0.107***
			[0.015]	[0.014]
- missing	-	-	0.030***	0.031***
			[0.007]	[0.007]
Years of experience	-	-	0.000***	0.000***
			[0.000]	[0.000]
Years of tenure	-	-	0.000	0.000
			[0.000]	[0.000]
Number of children by age category:				
- 0-5	-	-	0.000	0.001
			[0.002]	[0.002]
- 6-10	-	-	0.004*	0.005**
			[0.002]	[0.002]
- 11-15	-	-	-0.001	-0.000
			[0.002]	[0.002]
Part-time job (vs. full-time)	-	-	-0.016***	-0.016***
			[0.004]	[0.004]
Temporary job (vs. permanent)	-	-	-0.008***	-0.005
			[0.003]	[0.003]
Positive_pop	-	12.992***	-	11.156***
		[4.402]		[3.898]
Female workers proportion	-	-	-0.025	-0.023
			[0.019]	[0.019]
N. obs	67368	67368	67368	67368
R^2	0.028	0.066	0.226	0.241

Notes: * 0.1, ** 0.05, and *** 0.01 level of statistical significance. Cluster-robust S.E. are reported in brackets.
The treated group includes workers in non-essential sectors, whilst the control group represents workers in essential sectors. Model (1) includes no controls. Model (2) includes province and time fixed effects as well as the lagged weekly rate of contagion at the province level. Model (3) controls for the full set of individual and job characteristics. Model (4) is our preferred baseline model and controls for the full set of individual and job characteristics, for province and time fixed effects and for the lagged weekly rate of contagion at the province level. The estimated coefficients of all fixed effects are not reported, they are available from the authors upon request.
